# Integrative analysis of *Trichosanthes kirilowii* maxim formula granules’ anti-triple-negative breast cancer mechanism via network pharmacology, metabolomics, and molecular pharmacology

**DOI:** 10.3389/fphar.2026.1657396

**Published:** 2026-03-16

**Authors:** Yuzhen Gao, Yan Lu, Yaping Liu, Fen Liu, Jiaqi Zhang, Fei Xu, Zongwen Ji, Tian Fu, Shulong Shi, Shulong Jiang

**Affiliations:** 1 Clinical Medical Laboratory Center, Jining No. 1 People’s Hospital, Shandong First Medical University, Jining, Shandong, China; 2 Shandong University of Traditional Chinese Medicine, Jinan, Shandong, China; 3 Clinical Laboratory Medicine Department, Jining No. 1 People’s Hospital, Shandong First Medical University, Jining, Shandong, China; 4 Department of Endocrinology, Jining No. 1 People’s Hospital, Shandong First Medical University, Jining, Shandong, China; 5 Department of Vascular Surgery, Jining No. 1 People’s Hospital, Shandong First Medical University, Jining, Shandong, China; 6 Department of Respiratory and Critical Care Medicine, Jining No. 1 People’s Hospital, Shandong First Medical University, Jining, Shandong, China

**Keywords:** LC-MS/MS, metabolomics, network pharmacology, surface plasmon resonance, Trichosanthes kirilowii maxim, triple-negative breast cancer

## Abstract

**Background:**

Triple-negative breast cancer (TNBC) urgently needs effective therapies due to limited targeted options and unfavorable outcomes. We investigated *Trichosanthes kirilowii* Maxim formula granules (TKM) using integrated network pharmacology, metabolomics, and molecular pharmacology to clarify potential multi-target mechanisms relevant to TNBC.

**Methods:**

Liquid chromatography coupled with mass spectrometry was employed to identify the components of TKM formula granules. Network pharmacology-based prediction was used to uncover potential mechanisms by which TKM counteracts TNBC. Potential targets were identified, and pathway enrichment analysis was performed. Subsequently, TNBC cells and 4T1 tumor-bearing mice were used to verify the molecular mechanisms of TKM.

**Results:**

We identified 151 active compounds in TKM. Through network pharmacology analysis, 214 TNBC-related targets were found, with 28 core targets, including cell cycle and apoptosis regulators MYC, TP53, AKT1, CCND1, CASP3, PIK3CA, BCL2L1, and CDC42. The compound-target-pathway-disease network showed that schisandrin binds to many treatment targets with satisfactory docking performance, especially for MYC and AKT1. Experimentally, TKM was found to significantly promote apoptosis and induce G2/M-phase cell-cycle arrest in MDA-MB-231 cells. Western blot analysis showed that TKM suppressed PI3K/AKT and Wnt/β-catenin signaling pathways. Surface plasmon resonance experiment revealed that schisandrin binds to recombinant AKT1 with an equilibrium dissociation constant (K_D) of 1.525 × 10^−4^ M. According to untargeted metabolomics results, TKM can regulate amino acid, glycine, serine, and threonine metabolism, mineral absorption, protein digestion and absorption, central carbon metabolism, and arginine biosynthesis to exert therapeutic effects on TNBC. All findings were consistent with predicted targets and pathways.

**Conclusion:**

This study comprehensively explores the multi-target mechanisms of TKM against TNBC using network pharmacology, molecular pharmacology, and metabolomics approaches. These findings provide a foundation for future mechanistic investigations and may support the further preclinical development of TKM-based strategies for TNBC.

## Introduction

Triple-negative breast cancer (TNBC) is an aggressive subtype of breast cancer characterized by the absence of estrogen receptor (ER), progesterone receptor (PR), and human epidermal growth factor receptor 2 (HER2) expression, and is associated with early recurrence, visceral metastases, and poor prognosis (Hu et al., 2024; De Santis et al., 2024). For many patients, cytotoxic chemotherapy remains the main systemic option, and resistance and toxicity are common, highlighting the need for novel, mechanism-based therapeutic strategies, including a rational evaluation of complementary approaches (Xiong et al., 2024). Epigenetic reprogramming within the tumor microenvironment is also increasingly recognized as a key contributor to TNBC progression (Zhou and Yu, 2024).

Traditional Chinese medicines (TCM) are multi-component botanical formulations with diverse chemical constituents and multi-modal pharmacological actions (Chan, 2005; Zhao et al., 2023). Several TCM formulas and isolated constituents have shown preclinical activity in breast cancer models by modulating cell survival, apoptosis, angiogenesis, immunity, and inflammation (Zhang X. et al., 2021; Guo et al., 2022). However, their complexity makes it challenging to define active ingredients and mechanisms using conventional single-target pharmacology. Recent work has specifically reviewed TCM-based strategies for TNBC and breast cancer, including bibliometric and network pharmacology analyses that highlight key formulas, bioactive constituents, and immune/metabolic mechanisms (Wu et al., 2024; Feng et al., 2025; Song et al., 2025). Within this context, *Trichosanthes kirilowii* Maxim (Gua Lou) has long been used to treat phlegm retention, chest obstruction, and inflammatory conditions, and extracts or purified proteases derived from this species have demonstrated antitumor and anti-inflammatory activities in preclinical models (Ni et al., 2015; Park et al., 2019; Sun et al., 2020; Kim et al., 2022).

Network pharmacology introduces an innovative strategy rooted in systems biology and multi-directional pharmacology, serving as a contemporary system-oriented method for researching TCM (Zhang Y. Z. et al., 2021; Chen et al., 2023; Li et al., 2023). This aligns with the holistic function of TCM from a macro-level or overall regulatory perspective, providing insights into the molecular and pharmacological mechanisms of drugs. Network pharmacology, which relies on the “compound-protein/gene-disease” network, efficiently uncovers the molecular mechanisms of small molecules and excels at analyzing intricate systems (Li G. et al., 2022; Li X.et al., 2022). Methods of network pharmacology analysis are commonly employed to discover the active components and mechanisms of TCM as well as to explore new applications and innovative pharmaceuticals. This approach has been utilized to assess the effects of different TCMs, including dandelion (Rezaie et al., 2023), bitter gourd (Raina et al., 2016), and ginseng (Okuno et al., 2023). Metabolomics, a robust and comprehensive approach, is increasingly being used to explore disease pathogenesis and drug mechanisms (Cao et al., 2015). Metabolomics effectively evaluates the concentrations of low-molecular-weight molecules. Two types of mass spectrometry modes, positive and negative, are used to detect cellular metabolites. Based on their physical and chemical properties, metabolites can carry either a positive or negative charge. To obtain comprehensive information on untargeted metabolomics using liquid chromatography coupled with mass spectrometry (LC-MS), ions in both states of the study are scanned. Metabolomics has been applied extensively in cancer research (Buriani et al., 2012). Metabolites, the center of cellular metabolism, provide substrates and energy for biological processes and regulate the cell cycle, proliferation, and apoptosis (Ward and Thompson, 2012). Analysis of differentially accumulated metabolites can help identify tumor markers and elucidate the molecular mechanisms underlying antitumor drug resistance. Metabolomics provides substantial benefits for thoroughly exploring the mechanisms of TCM and clarifies the connections between metabolites and metabolic pathways in both pathological and physiological states (Li et al., 2021). Metabolomics can help identify the active components in TCM and discover their biological targets. Comparative analysis of metabolomic data from TCM treatment and control groups revealed metabolic changes induced by TCM treatment, thereby elucidating its mechanism. This will enhance understanding of TCM’s therapeutic principles and support its clinical application (Wei et al., 2022). However, to date, no study has reported the molecular mechanisms of TKM’s anti-cancer effects in triple-negative cancer based on network pharmacology or metabolomics research.

This study integrated network pharmacology with molecular biology experiments to comprehensively analyze and explore the possible molecular mechanisms underlying TKM inhibition of TNBC. Simultaneously, we applied untargeted metabolomics technology to reveal the suppression of TNBC cell proliferation by TKM at the metabolic pathway level. Previous studies have combined network pharmacology and metabolomics to investigate other TCMs in TNBC, usually linking complex formulas to PI3K/AKT and metabolic pathways at a correlative level (for example, dandelion-based extracts and Xihuang Pill). In this work, beyond reproducing such an integrative workflow, we highlight schisandrin as one of the key active components of TKM and provide experimental evidence that it directly binds AKT1 by surface plasmon resonance and exerts AKT1-dependent antiproliferative effects in TNBC cells, thereby offering a more mechanistically anchored compound-target example for TKM.

## Materials and methods

### Chemicals and reagents

The TKM formula granules (Batch Number: 2307003C) were purchased from China Resources Sanjiu Medical and Pharmaceutical Co., Ltd. According to the national standard (YBZ-PFKL-2021173), the corresponding TKM formula granules were weighed out and dissolved in sterile water, passed through a 0.22 μm filter, and configured into a 2000 mg/mL TKM original solution.

### Qualitative HPLC-Orbitrap-MS/MS analysis of TKM

For qualitative chemical profiling, 0.10 g of TKM granules was extracted with 1.0 mL of 80% methanol in water (v/v), vortexed, centrifuged, and the supernatant was filtered for LC–MS/MS analysis.

Chromatographic separation was performed on a Thermo UPLC system using a reversed-phase column and a conventional water-acetonitrile gradient containing 0.1% formic acid at a flow rate of 0.3 mL/min; detailed gradient conditions are provided in the Supplementary Methods.

High-resolution MS detection was carried out on a Q Exactive Orbitrap mass spectrometer operated in positive/negative electrospray mode, and data acquisition and processing were performed with vendor software as described in the Supplementary Materials.

In addition, the content of schisandrin in the TKM granules was quantified by HPLC using UV detection and an external calibration curve constructed from a schisandrin reference standard. The chromatographic conditions were optimized based on the qualitative HPLC-Orbitrap-MS/MS analysis, and the measured schisandrin content in the TKM preparation is summarized in the Supplementary Materials.

### Network pharmacology study

For TKM, according to the results of HPLC-Orbitrap-MS/MS analysis, LC–MS features with an mzCloud Best Match ≥70 were retained as candidate constituents (Bi et al., 2020). To enrich for orally bioavailable, drug-like compounds, predicted gastrointestinal absorption and oral drug-likeness were evaluated using SwissADME; components with high GI absorption and satisfying at least two commonly used drug-likeness filters (Lipinski, Ghose, Veber, Egan, Muegge) were considered active ingredients for subsequent analyses (Daina et al., 2017). The corresponding targets were obtained by querying the SwissTargetPrediction database (Daina et al., 2017). In our research, we gathered known targets associated with breast cancer from the GeneCards database by utilizing the keyword “breast cancer” (Stelzer et al., 2016). The dataset GSE139038 comprised 41 patients alongside 17 controls sourced from the GPL27630 platform. We analyzed microarray datasets of differentially expressed mRNAs between the normal group and TNBC patients using the “limma” package in R. Genes were deemed pathogenic and related to TNBC if they had an adjusted p-value of less than 0.05 and an absolute log2 (fold change) greater than 2. Through the intersection of TKM and TNBC targets, we identified shared targets as potential therapeutic targets for TKM in the context of TNBC. The gene characteristics of these therapeutic targets were determined using the DisGeNet database (Li G. et al., 2022). Justification of thresholds: For MS/MS library matching, an mzCloud best-match score ≥70 was chosen to balance sensitivity and specificity and to reduce false positives from ambiguous spectra (Bi et al., 2020). Candidate TKM constituents were further required to have high predicted gastrointestinal absorption and to satisfy at least two standard oral drug-likeness rules (Lipinski, Ghose, Veber, Egan, Muegge) in SwissADME, in order to enrich for orally bioavailable, pharmaceutically tractable compounds. For the transcriptomic dataset (GSE139038), we applied an absolute log2 (fold change) > 2 together with an adjusted p-value <0.05 to focus on robust gene-expression changes. For KEGG pathway enrichment, Benjamini–Hochberg false discovery rate (FDR) correction was used, and pathways with FDR-adjusted p < 0.05 were considered significantly enriched.

The therapeutic targets' protein-protein interaction relationships were sourced from the STRING database (https://cn.string-db.org/), while Cytoscape software facilitated the integration, visualization, and analysis of biological networks. In the protein-protein interaction network, the core genes, which were identified as potential therapeutic targets, were prioritized using a comprehensive evaluation of various centrality measures. These measures included degree centrality, betweenness centrality, closeness centrality, Eigenvector centrality, and the local average connectivity-based method. The prioritization was performed using the CytoNCA plugin.

Gene Ontology (GO) and Kyoto Encyclopedia of Genes and Genomes (KEGG) enrichment analyses were conducted to elucidate the biological relevance of therapeutic targets. For the biological process within the GO enrichment analysis, the ClueGo plugin was utilized, while the R package clusterProfiler was employed for KEGG pathway enrichment analysis (adjusted p-value <0.05). Ultimately, a network encompassing compounds, targets, pathways, and diseases was created with the help of Cytoscape software.

Molecular configurations of the core targets identified were ascertained through the Protein Data Bank (http://www.rcsb.org/) database, stored in Protein Data Bank format, and subsequently uploaded to AutoDockTools software (Velankar et al., 2021). In addition, the structure of the key candidate constituent for docking was sourced from the PubChem database and imported into AutoDockTools in the MOL2 format. PyMol, a robust visualization tool for proteins and molecules, was employed to modify the proteins by eliminating the original ligands and water molecules, as well as correcting the protein structures. A genetic algorithm based on Lamarckian principles was utilized for the calculation of binding energies.

### Cell cultures

The cell lines for TNBC, specifically MDA-MB-231, SUM-159, and 4T1, were obtained from the Cell Bank of the Chinese Academy of Sciences located in Shanghai, China. These cell lines were maintained in complete DMEM medium (Gibco, NY, United States), which was supplemented with 10% fetal bovine serum (Gibco, NY, United States) and 1% penicillin-streptomycin. They were kept in a cell culture incubator (Model 3111, Thermo, CA, United States) under a humidified environment with 5% CO2 at a temperature of 37 °C.

### Cell proliferation assays

Cell viability was assessed utilizing the Cell Counting Kit-8 (Dojindo, Japan), in accordance with the protocol provided by the manufacturer. The cells were plated in 96-well plates at a concentration of 3000 cells per well. After an overnight incubation period, various concentrations of TKM were applied for either 24 or 48 h, with a blank control group included in the setup. The supernatant was subsequently discarded, and 100 µL of DMEM (without fetal bovine serum) combined with 10 μL of Cell Counting Kit-8 solution (Dojindo, Japan) was introduced into each well. After incubating for 30 min at 37 °C in the dark, absorbance readings were taken at 450 nm with a plate reader. The IC50 values of TKM were determined for 24 and 48 h across all 3 cell lines.

Cells undergoing logarithmic growth were plated in a 6-well dish at a density of 1500 cells per well and incubated overnight for the purpose of the colony formation assay. Following this, they were exposed to various concentrations of TKM for a duration of 2 weeks, with the media being changed every 3 days. After incubation, the cells were washed twice with PBS, fixed with paraformaldehyde for 30 min, and stained with 0.1% crystal violet solution for 30 min. The plates were dried overnight.

### Cell apoptosis and cycle assays

MDA-MB-231 cells were treated with TKM (0, 7.5, 15, or 30 mg/mL) for 24 h. The Annexin V APC Apoptosis Detection Kit (Biogems, United States) and Cell Cycle Assay Kit (Elabscience, China) were used to detect the effect of TKM on MDA-MB-231 cell apoptosis and the cell cycle, following the manufacturer’s instructions.

### Animal treatment for tumor-bearing experiments

The Ethics Committee for Animal Experiments at Jining First People’s Hospital authorized the experimental protocol (NO. JNRM-2024-DW-075). Antitumor efficacy of TKM was investigated using 20 female BALB/c mice that had 4T1 tumors. Following a 5-day acclimatization period, each mouse received a subcutaneous injection of 1.0 × 10^6^ cells in PBS into the right armpit. The subsequent day, the mice with tumors were divided randomly into four groups: one control group (TC) receiving normal saline (0.2 mL/day), a doxorubicin (DOX) group (administered 2 mg/kg via tail vein injection daily starting from day four), and two groups treated with TKM at doses of 0.59 and 4.68 g/kg/day, beginning on the second day. The weight and volume of each tumor-injected mouse were recorded every other day from day four onwards. TKM treatment continued for a total of 14 days. Twelve hours after the last drug administration, the mice were euthanized, and the tumors were excised for further examination. Following the measurements, the tumor tissues were utilized for untargeted metabolomics analysis.

For untargeted metabolomics, differential metabolites were screened using a combination of orthogonal partial least squares–discriminant analysis (OPLS-DA) and univariate testing. Features with a variable importance in projection (VIP) > 1 and nominal p < 0.05 (two-sided t-test) were considered significantly altered, without applying an explicit fold-change cut-off. Benjamini–Hochberg FDR correction was applied at the level of KEGG pathway enrichment to control for multiple testing across pathways.

To validate amino-acid metabolism at the metabolite level, targeted LC–MS/MS analysis of amino acids and related metabolites in 4T1 TNBC cells treated with TKM or vehicle was performed by a commercial service provider (Shanghai Applied Protein Technology Co., Ltd., Shanghai, China). Briefly, 4T1 cell pellets were extracted with ice-cold methanol/acetonitrile (1:1, v/v) containing isotope-labeled internal standards, followed by low-temperature ultrasonication, protein precipitation at −20 °C, centrifugation, vacuum drying, and reconstitution in acetonitrile/water (1:1, v/v). The supernatants were analyzed on an Agilent 1290 Infinity UHPLC system coupled to an AB Sciex 6500+ QTRAP mass spectrometer equipped with a UPLC BEH Amide column (2.1 × 100 mm, 1.7 μm). Amino acids and related metabolites were separated using a hydrophilic-interaction liquid chromatography gradient with acetonitrile containing 0.1% formic acid and aqueous 10 mmol/L ammonium acetate containing 0.1% formic acid as mobile phases, and detected in positive/negative electrospray multiple-reaction monitoring mode. A pooled quality-control sample was injected at regular intervals to monitor system stability. Chromatographic peaks were integrated using MultiQuant software and metabolites were identified by comparison with authentic standards. Metabolites with a coefficient of variation <15% in quality-control samples were retained for statistical analysis, and differences between TKM- and vehicle-treated groups were assessed using Student’s t-test with p < 0.05 considered statistically significant.

### Western blotting

Cell lysates were obtained through the use of RIPA assay lysis buffer. To determine protein concentration, a BCA assay kit was employed. The protein samples were subjected to separation via SDS-PAGE at a voltage of 70 V for 2 h, followed by transfer to PVDF membranes. These membranes were then incubated with 5% nonfat milk for 2 h at room temperature to block. After blocking, the membranes were incubated overnight at 4 °C with the following primary antibodies: PI3K, p-PI3K, AKT, p-AKT, β-catenin, and AKT1, each diluted at a ratio of 1:1000. After washing with TBST buffer, the membranes were treated with HRP-conjugated goat anti-rabbit antibody (1:20,000) for 2 h at room temperature. The protein bands were detected using the ECL kit on the ChemiDoc™ machine (BIO-RAD, United States). β-actin and vinculin acted as control proteins, and the expression levels of the other proteins were evaluated in relation to them.

### AKT1 knockdown assay

An oligonucleotide synthesized chemically, which encodes a short hairpin RNA aimed at AKT1 (5′-GCT​ACT​TCC​TCC​TCA​AGA​A-3′), was introduced into a pPLKO.1-PURO vector (AZENTA, China). Similarly, a control plasmid was developed. Following the manufacturer’s instructions, the purified expression plasmid containing short hairpin RNA specific for AKT1 and the control plasmid were introduced into MDA-MB-231 cells using Lipofectamine™ 3000 (Invitrogen, United States). Two days post-transfection, drug selection commenced with 2 μg/mL puromycin (Sigma-Aldrich), leading to the harvesting and expansion of colonies into cell lines.

### Surface plasmon resonance assay

Surface plasmon resonance assays were performed to evaluate the binding of schisandrin to recombinant AKT1 protein. In this study, the recombinant AKT1 protein was obtained from Med Chem Express. It was then prepared in a sodium acetate buffer at pH 4.0, achieving a concentration of 20 μg/mL. AKT1 protein was immobilized onto a CM5 chip using amino coupling. Two-fold serial dilutions of schisandrin (100, 50, 25, 12.5, 6.25, and 3.125 μM) were prepared in PBS-P solution containing 5% DMSO to determine the K_D of AKT1 towards schisandrin. The K_D value was calculated using Biacore T100 Kinetics Summary Software (version 1.0.1).

### Statistical analysis

All data are expressed as the mean ± standard deviation, and each experiment was conducted independently on three occasions. Significant differences were assessed using the Student’s t-test (*p < 0.05).

## Results

### TKM inhibits the proliferation of TNBC cells *in vitro* and *in vivo*

**FIGURE 1:**
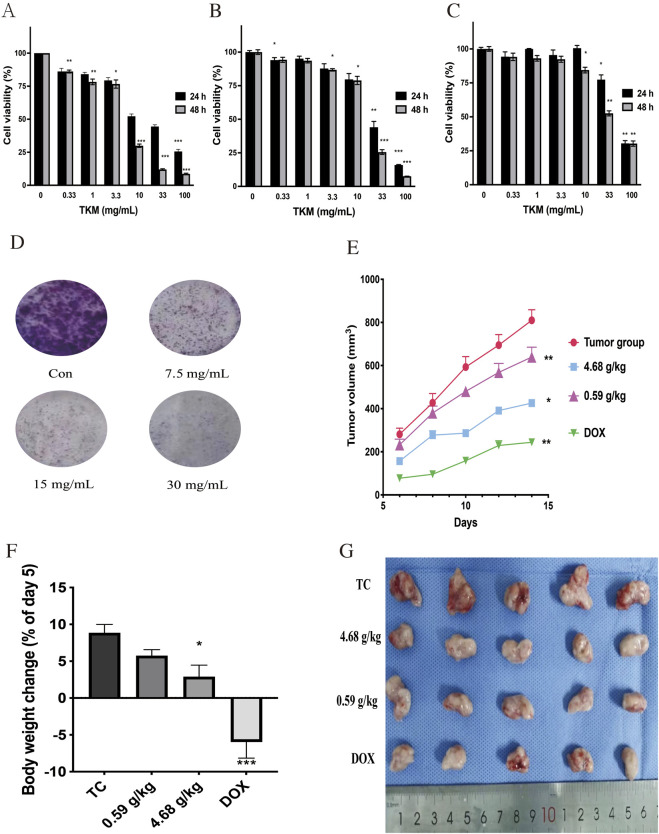
TKM-inhibited proliferation of TNBC cells and growth of mouse 4T1 breast tumors. **(A–C)** TNBC cells (MDA-MB-231, SUM-159, and 4T1) were treated with different concentrations of TKM for 24 and 48 h to examine the cell viability. **(D)** MDA-MB-231 cells were treated with different concentrations of TKM and then cultured in a fresh medium for 2 weeks to evaluate the colony formation abilities. **(E)** The volumes of 4T1 homograft tumors were measured on day 15. **(F)** Body weights of mice were monitored during the experiment, and body weight change is presented as % of the day 5 value (day 5 indicates the end of the 5-day acclimation period after arrival and was used as the normalization baseline, set as 100%). **(G)** Photographs of TKM- or doxorubicin-treated homografts derived from BALB/c mice are shown. Mean ± SD was calculated for all experiment values. Asterisks indicate statistically significant differences compared with the corresponding control group (0 mg/mL/blank control for the *in vitro* assays in panels **(A–D)** and the tumor control (TC) group for the *in vivo* tumor measurements in panels E–F; p < 0.05, Student’s t-test).

To explore the impact of TKM on TNBC cells, MDA-MB-231, SUM-159, and 4T1 cell lines were subjected to various concentrations of TKM over different time periods, and cell viability was assessed using Cell Counting Kit-8. Figures 1A–C illustrate that TKM treatment led to a dose- and time-dependent inhibition of growth in all three TNBC cell lines. Additional results from the colony formation assay further supported the anti-TNBC efficacy of TKM *in vitro* (Figure 1D).

To verify the impact of TKM on tumor growth *in vivo*, BALB/c mice received injections of 4T1 cells, followed by daily oral administration of TKM at doses of 0.59 and 4.68 g/kg. As illustrated in Figure 1E, the tumor control group displayed the largest average tumor volume, measuring 1.070 cm^3^. In contrast, treatment with TKM resulted in a significant inhibition of tumor progression, with reductions of 66.01% and 76.84% observed at the dosages of 0.59 and 4.68 g/kg, respectively. Additionally, throughout the treatment duration, the mice did not show any notable fluctuations in body weight (Figure 1F), suggesting that TKM did not have any toxic effects. A photograph of the tumor tissue taken at the end of the treatment is displayed in Figure 1G.

### Network pharmacology prediction of TKM for TNBC

The chemical compounds of TKM were identified using LC-MS analysis. Supplementary Figure S1 shows the LC-MS total ion chromatogram of TKM. Compared to the mzCloud database, we identified 810 putative compounds, 293 of which had an mzCloud best-match score higher than 70 (Supplementary Table S1). In total, 151 compounds showed high GI absorption and had more than two drug-like properties, including Lipinski, Ghose, Veber, Egan, and Muegge, and thus, 1164 targets of the 151 compounds were selected for further investigation (Supplementary Figure S2).

**FIGURE 2:**
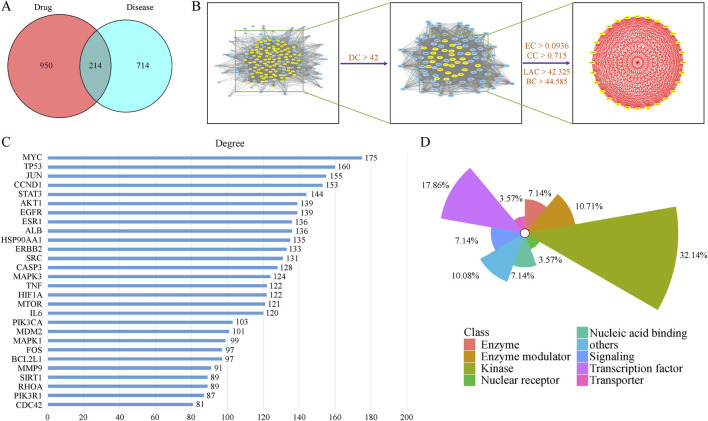
PPI network analysis and target prediction for TKM in TNBC. **(A)** Venn diagram showing the intersection between predicted TKM targets and TNBC-related genes. **(B)** Protein–protein interaction network with node centrality metrics (degree, betweenness, eigenvector, closeness, and local average connectivity) used to identify core therapeutic targets. **(C)** The 28 core therapeutic targets ranked by degree. **(D)** Gene attribute categories of the core targets based on DisGeNET annotation. The original high-resolution image for panel **(B)** is provided in the Supplementary Materials.

By mining the Gene Expression Omnibus (GEO) database, 950 genes satisfying the filtering conditions were identified. Additionally, 714 known breast cancer-related genes were identified in the GeneCards database. By merging and deduplicating these two datasets, 1450 breast cancer-related targets were obtained (Supplementary Figure S3). A total of 214 intersectional targets were identified between the disease and drug targets (Figure 2A). These intersectional targets are regarded as therapeutic targets in TKM-treated TNBC. By querying the DisGeNet database, these treatment targets were observed to involve numerous attributes, which reflects TKM’s multi-faceted and multi-dimensional regulation of breast cancer.

**FIGURE 3:**
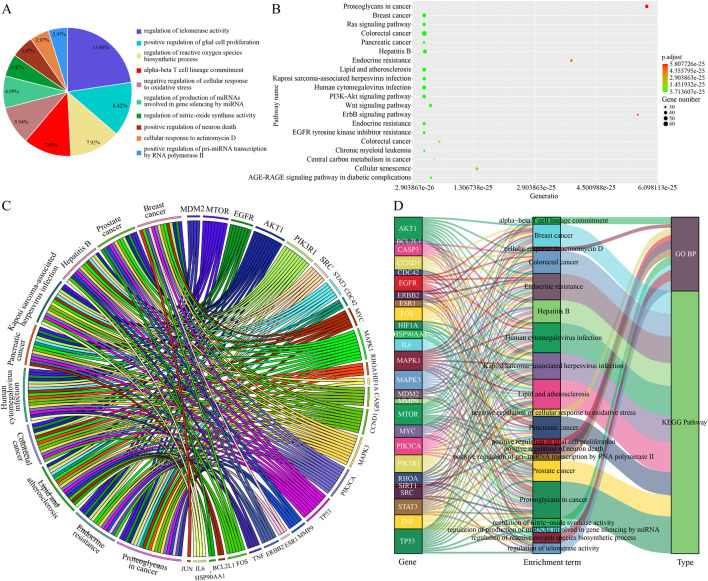
GO biological process and KEGG pathway enrichment analyses of the core therapeutic targets of TKM in TNBC. **(A)** Pie chart showing the top 10 enriched GO biological process (GO BP) terms for the 28 core targets. **(B)** Bubble plot of KEGG pathway enrichment; the x-axis represents the gene ratio, dot size indicates the number of genes, and dot color reflects the adjusted p value. **(C)** Circos plot illustrating the relationships between the 28 core targets and representative KEGG pathways. **(D)** Sankey diagram displaying the associations among core genes, enriched GO BP terms, and KEGG pathways.

**FIGURE 4:**
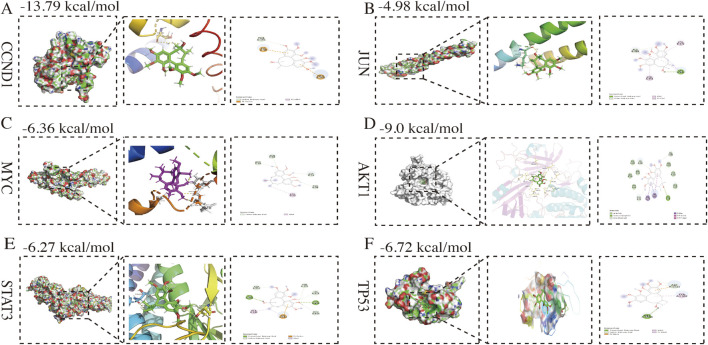
Molecular docking pattern of schisandrin with target proteins. **(A)** CCND1; **(B)** JUN; **(C)** MYC; **(D)** AKT1; **(E)** STAT3; **(F)** TP53.

Through computational analysis of degree centrality, betweenness centrality, eigenvector centrality, closeness centrality, and local average connectivity (Figure 2B), 28 core therapeutic targets were identified. They are shown in Figures 2C,D. Using the ClueGo plugin for GOBP functional annotation (Supplementary Figure S4.), we observed that the primary biological processes were “regulation of telomerase activity, positive regulation of glial cell proliferation, regulation of reactive oxygen species biosynthetic process, alpha-beta T cell lineage commitment, and negative regulation of cellular response to oxidative stress” (Figure 3A). KEGG pathway enrichment analysis was conducted using the R language package Cluster Profiler. The results indicate that TKM may help treat TNBC by “proteoglycans in cancer, Ras signaling pathway, PI3K/AKT signaling pathway, Wnt/β-catenin signaling pathway, and ErbB signaling pathway” (Figure 3B). GO, BP, and KEGG pathway analyses revealed that “MYC, TP53, JUN, CCND1, STAT3, and AKT1” may facilitate the treatment (Figures 3C,D). Subsequently, using the Cytoscape software, we established a compound-target-pathway-disease network (Supplementary Figure S5), revealing that schisandrin binds to more treatment targets (Supplementary Table S2), suggesting that it may be the key bioactive constituent for breast cancer treatment. The molecular docking performances were satisfactory (Figure 4).

### Effect of TKM on the cell cycle and apoptosis of TNBC cells

**FIGURE 5:**
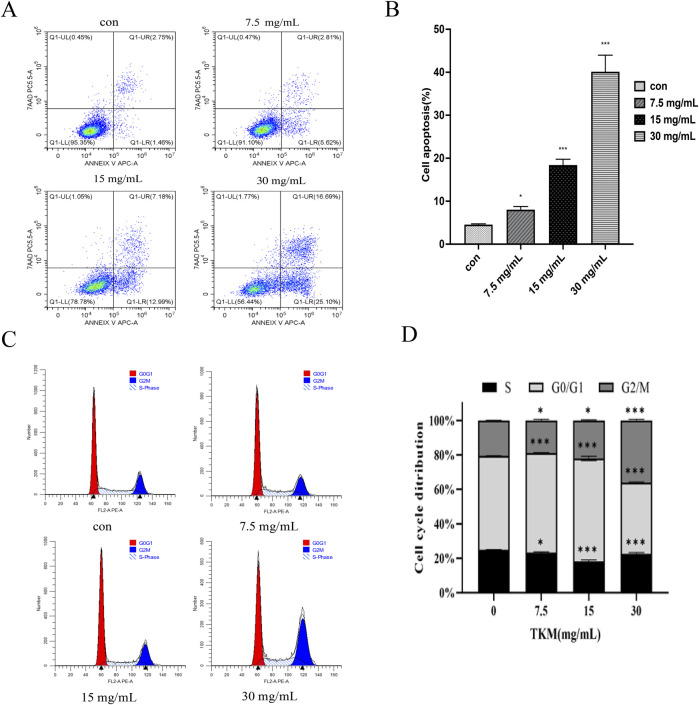
TKM induces apoptosis and cell cycle arrest in MDA-MB-231 cells. **(A,B)** Flow-cytometry analysis for apoptosis of MDA-MB-231 cells treated with different concentrations of TKM. **(C,D)** Flow-cytometry analysis for cell cycle distribution of MDA-MB-231 cells treated with different concentrations of TKM. Asterisks indicate statistically significant differences between each TKM-treated group and the 0 mg/mL control (p < 0.05, Student’s t-test).

Through network pharmacology analysis, we identified 28 key targets related to TNBC proliferation and survival and then focused our experimental validation on representative functional readouts rather than reiterating the full *in silico* workflow. Consistent with these predictions, TKM reduced the viability of MDA-MB-231, SUM-159, and 4T1 cells in a dose- and time-dependent manner, suppressed colony formation, induced apoptosis, and caused G2/M-phase cell-cycle arrest in MDA-MB-231 cells, indicating a robust antiproliferative effect on TNBC cells (Figure 5).

### Validation of signal pathway and target

**FIGURE 6:**
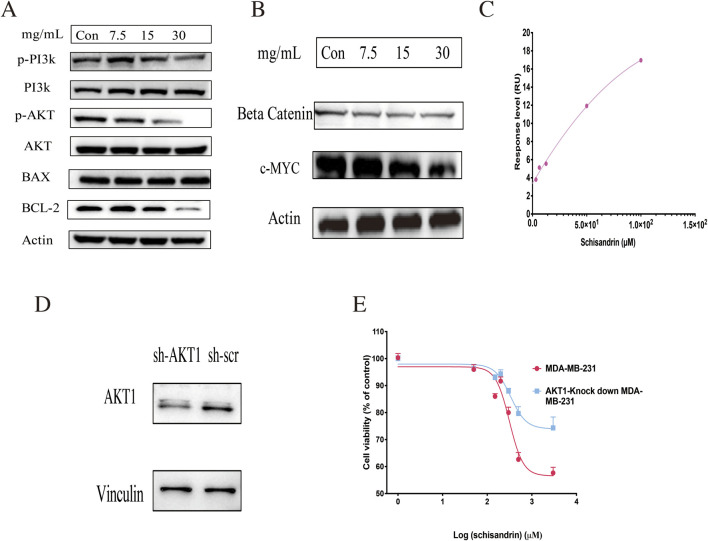
Validation of cell signaling pathways and the AKT1 target. **(A,B)** Effects of TKM on PI3K/AKT and Wnt/β-catenin signaling pathways and their downstream effectors in MDA-MB-231 cells, as assessed by Western blotting of the indicated signaling proteins, including the pro-apoptotic protein BAX, the anti-apoptotic protein BCL2, and the oncogenic transcription factor c-MYC. **(C)** Surface plasmon resonance analysis of schisandrin binding to recombinant AKT1 protein. **(D)** Knockdown efficiency of AKT1 in MDA-MB-231 cells. **(E)** Effects of schisandrin on the proliferation of control and AKT1-knockdown MDA-MB-231 cells. Data are presented as mean ± SEM of three independent experiments.

KEGG pathway analysis indicated enrichment of the PI3K/AKT and Wnt/β-catenin pathways, suggesting that these signaling axes may be involved in the cellular mechanisms by which TKM inhibits TNBC. To functionally validate these predictions, we performed Western blotting in MDA-MB-231 cells. TKM reduced the phosphorylation of PI3K and AKT and decreased β-catenin levels, and, importantly, it increased the pro-apoptotic protein BAX while decreasing the anti-apoptotic protein BCL2, thereby elevating the BAX/BCL2 ratio. In parallel, TKM treatment markedly reduced the expression of the oncogenic transcription factor c-MYC, a key downstream effector of Wnt/β-catenin signaling (Figures 6A,B). These findings indicate that TKM not only modulates PI3K/AKT and Wnt/β-catenin at the level of core kinases but also suppresses their downstream prosurvival and proliferative outputs in TNBC cells. Network pharmacology analysis identified schisandrin as a candidate active component, and molecular docking suggested that it can interact with AKT1 (Figure 4D). Surface plasmon resonance experiments provided additional support that schisandrin binds to recombinant AKT1 protein with a K_D of 1.525 × 10^−4^ M (Figure 6C). Moreover, schisandrin inhibited the proliferation of control MDA-MB-231 cells more strongly than that of AKT1-knockdown cells (Figures 6D,E), supporting AKT1 as one of the functional effectors engaged by schisandrin in this system.

### Untargeted and targeted metabolomic analyses

**FIGURE 7:**
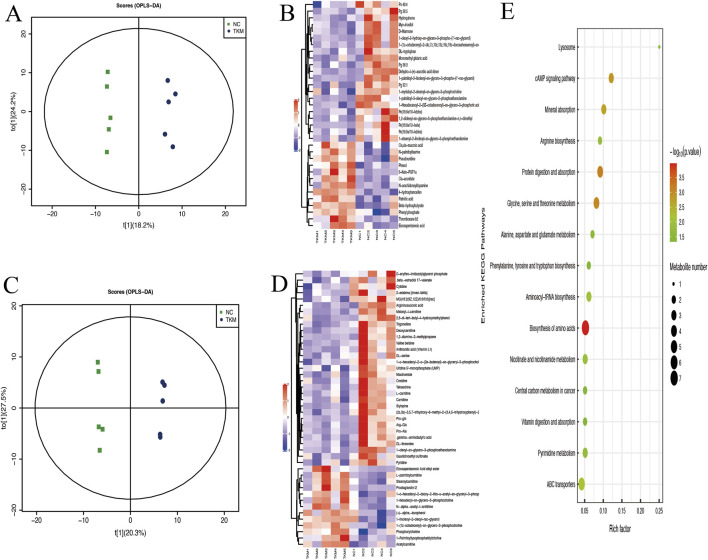
Untargeted metabolomics of tumor tissues after TKM treatment. **(A,C)** OPLS-DA score plots separating control (NC; untreated) and TKM-treated (TKM) groups in negative **(A)** and positive **(C)** ion modes; model performance: negative, R^2^ = 0.992 and Q^2^ = 0.570; positive, R^2^ = 0.997 and Q^2^ = 0.563. **(B,D)** Hierarchical-clustering heatmaps showing standardized abundances of differential metabolites (VIP >1 and two-sided t-test p < 0.05) in negative **(B)** and positive **(D)** ion modes. **(E)** Pathway-level enrichment of altered metabolites, highlighting amino acid biosynthesis; glycine, serine and threonine metabolism; mineral absorption; protein digestion and absorption; and central carbon metabolism in cancer.

To determine if TKM affects TNBC by influencing the metabolism of tumor tissues, an untargeted metabolomics strategy utilizing LC‒Q-TOF/MS was employed to examine the metabolic variations in tumor tissues treated with and without TKM. A supervised OPLS-DA model was created (Figures 7A,C) to pinpoint the elements responsible for group distinctions. The R^2^ values for the positive and negative MS ion modes in the OPLS-DA model were recorded at 0.997 and 0.992, while the Q^2^ values were 0.563 and 0.57, respectively. Differential metabolites were identified from all variables that fulfilled the criteria of VIP >1 in OPLS-DA and p < 0.05 based on the t-test (Supplementary Tables S4, 5). We then performed hierarchical clustering of metabolites potentially influenced by TKM across both positive and negative ion modes (Figures 7B,D). Furthermore, the pathways of the differential metabolites were analyzed as potential target pathways (Figure 7E). Within this context, representative metabolic pathways include the biosynthesis of amino acids; glycine, serine, and threonine metabolism; mineral absorption; and protein digestion and absorption. Alterations in central carbon metabolism in cancer, which aligned with the results of network pharmacology, were also detected. KEGG pathway enrichment of these differential metabolites was analyzed using Benjamini–Hochberg FDR correction, and pathways with FDR-adjusted p < 0.05 were regarded as significantly altered.

To validate the tumor-tissue metabolomics at the metabolite level in an independent system, we additionally performed targeted LC–MS/MS profiling of more than 80 amino acids and related metabolites in 4T1 TNBC cells treated with TKM versus vehicle. Quality-control samples showed a mean coefficient of variation of approximately 4.8%, with more than 95% of quantified metabolites exhibiting coefficient of variation (CV) < 15%, indicating good analytical robustness. Approximately 30 metabolites were significantly altered (p < 0.05) in TKM-treated cells, including decreases in glycine, threonine, phosphoserine, and asparagine and increases in glutamine and aspartic acid, in line with the amino-acid metabolism pathways highlighted by the untargeted tumor metabolomics. These targeted data further support amino-acid metabolic reprogramming as an important downstream effect of TKM in TNBC and are provided in detail in the Supplementary Table S6.

## Discussion

This study combined network pharmacology, metabolomics, and molecular and animal experiments to explore the antitumor mechanisms of TKM in TNBC. The main findings are as follows: (1) TKM inhibited TNBC cell proliferation and 4T1 tumor growth, extending previous evidence of its antitumor activity (Park et al., 2019). (2) Network pharmacology highlighted PI3K/AKT, Wnt/β-catenin, and amino-acid metabolism as candidate regulatory axes. (3) Molecular assays supported the notion that TKM modulates these pathways and promotes apoptosis and cell-cycle arrest. (4) Untargeted tumor-tissue metabolomics, together with targeted amino-acid profiling, indicated perturbation of amino-acid metabolism. Taken together, these data generate and preliminarily validate a mechanistic framework for how TKM may exert antitumor effects in TNBC, but they remain hypothesis-generating rather than definitive.

Compared with earlier network pharmacology–metabolomics studies of TCMs in TNBC, which mainly infer multi-target actions from *in silico* predictions and pathway enrichment, our work provides experimental support for a schisandrin–AKT1 axis. Schisandrin, highlighted as a hub compound in the TKM–target network and predicted to interact with AKT1, bound recombinant AKT1 in surface plasmon resonance assays and showed reduced antiproliferative activity in AKT1-knockdown MDA-MB-231 cells, consistent with AKT1 functioning as one effector of TKM in TNBC. LC–MS/MS profiling combined with drug-likeness and absorption, distribution, metabolism, and excretion (ADME) filtering and compound–target–pathway analyses also pointed to schisandrin as a key candidate active constituent within the multicomponent TKM. However, we have not yet performed comprehensive functional dissection (e.g., schisandrin alone versus complete TKM, add-back or occlusion designs) *in vitro* or *in vivo*. Accordingly, we regard schisandrin as a mechanistically anchored example of a bioactive constituent rather than the sole or definitively “main” driver of TKM efficacy, and we view such rescue studies as an important direction for future work.

At the cellular level, TKM reduced the viability and colony formation of MDA-MB-231, SUM-159, and 4T1 cells, induced apoptosis, and caused G2/M-phase cell-cycle arrest, indicating a robust antiproliferative effect. Consistent with network predictions, TKM decreased PI3K and AKT phosphorylation, reduced β-catenin, increased BAX, decreased BCL2, and lowered c-MYC expression. These findings suggest that TKM attenuates PI3K/AKT- and Wnt/β-catenin-driven pro-survival signaling and shifts the balance toward apoptosis in TNBC cells. Tumor-tissue metabolomics indicated that TKM modulates amino-acid biosynthesis, glycine/serine/threonine metabolism, and central carbon metabolism, and targeted LC–MS/MS in 4T1 cells confirmed significant changes in multiple amino acids and related metabolites, supporting amino-acid metabolic reprogramming as an important downstream effect of TKM. These observations align with reports that serine/glycine metabolism supports TNBC growth and metastatic behavior (Qu et al., 2022; Zhou et al., 2024). In our study, TKM also inhibited MDA-MB-231 invasion and migration in Transwell® assays (Supplementary Figure S6), providing preliminary functional support for an impact on invasive behavior. Collectively, these data suggest that TKM exerts its antitumor effects, at least in part, by concomitantly dampening oncogenic signaling and rewiring amino-acid metabolism in TNBC.

Using standard body-surface-area allometry (FDA Km method), the high TKM dose used in the 4T1 model (4.68 g/kg/day) corresponds to a human-equivalent dose of approximately 22.8–26.6 g/day for a 60–70-kg adult. Clinically, TKM is usually prescribed at about 9–15 g/day per adult (Chinese Pharmacopoeia Commission, 2020), indicating that our regimen is supratherapeutic but still within roughly 1.5–1.8-fold of the upper clinical dose. We therefore regard it as an upper-bound proof-of-concept exposure that brackets the efficacy window while remaining clinically proximate, and it highlights the need for dedicated pharmacokinetic, dose-ranging toxicology, and standardization studies before any extrapolation to human dosing.

Although the present work focused on efficacy, translational advancement will require establishing an adequate *in vivo* safety margin for TKM. In line with oncology-specific nonclinical principles, future studies should include dose-ranging, repeat-dose toxicity studies in appropriate species, together with clinical chemistry/hematology and targeted histopathology, to define the maximum tolerated dose (MTD) and no-observed-adverse-effect level (NOAEL) and to inform formulation and therapeutic index (ICH, 2009). Given TKM’s multicomponent nature and the risk of herb–drug interactions, early incorporation of *in vitro* cytochrome P450 and transporter assays (e.g., CYP3A/2D6, P-gp/OATP) is warranted to de-risk drug–drug interactions (DDIs) with standard TNBC regimens (U.S. Food and Drug Administration, 2020). Where feasible, tumor-bearing *in vivo* models should integrate safety readouts alongside efficacy endpoints to approximate benefit–risk prior to first-in-human evaluation.

Several limitations of this study should be acknowledged. First, all data are preclinical and were generated using a restricted set of experimental models (three TNBC cell lines and a single syngeneic 4T1 tumor model). These systems cannot capture the full molecular and clinical heterogeneity of TNBC, and our findings should therefore be interpreted as mechanistic proof-of-concept rather than as evidence of clinical efficacy. Second, many of the targets and pathways identified are, at least in part, based on *in silico* network pharmacology predictions, and the proposed mechanisms are provisional and require confirmation in additional *in vitro* and *in vivo* studies, including more diverse TNBC models. Third, clinical pharmacokinetic, pharmacodynamic, and formal toxicology data for TKM in oncology are lacking; as a result, the safety margins, drug–drug interaction potential, and optimal scheduling of TKM, either alone or in combination with existing therapies, remain to be defined. Finally, although we attempted to rationalize dose selection using traditional usage ranges and allometric scaling, the relatively high experimental doses used here may be difficult to implement directly in clinical practice, and future studies will need to identify minimally effective doses and biomarkers of response to guide rational translation.

To place TKM within the current TNBC treatment landscape, it is important to distinguish it from established therapies. At present, standard-of-care management of TNBC relies primarily on multi-agent cytotoxic chemotherapy (for example, anthracycline- and taxane-based regimens). PARP inhibitors are used mainly in patients with germline or tumor BRCA1/2 mutations or other homologous-recombination defects, and immune checkpoint inhibitors targeting PD-1/PD-L1 are employed in selected patients in combination with chemotherapy. In contrast, TKM is a multicomponent herbal formula with incompletely defined pharmacology and no robust clinical evidence in TNBC to date. In its current state, TKM should therefore be viewed as an experimental, potentially complementary approach that might in future be evaluated as an adjunct to standard therapies to modulate tumor biology, metabolism, or treatment tolerance, rather than as an alternative to proven chemotherapeutic, targeted, or immunotherapeutic regimens. Before any such combinations are considered, potential pharmacokinetic and pharmacodynamic herb–drug interactions, as well as the risk of attenuating efficacy or exacerbating toxicity, will need to be rigorously investigated in dedicated preclinical and clinical studies. This perspective is consistent with recent reviews and mechanistic analyses of TCM in TNBC and breast cancer, which similarly emphasize its role as an adjunctive, multi-target strategy rather than a replacement for standard regimens (Wu et al., 2024; Feng et al., 2025; Song et al., 2025).

## Conclusion

**FIGURE 8:**
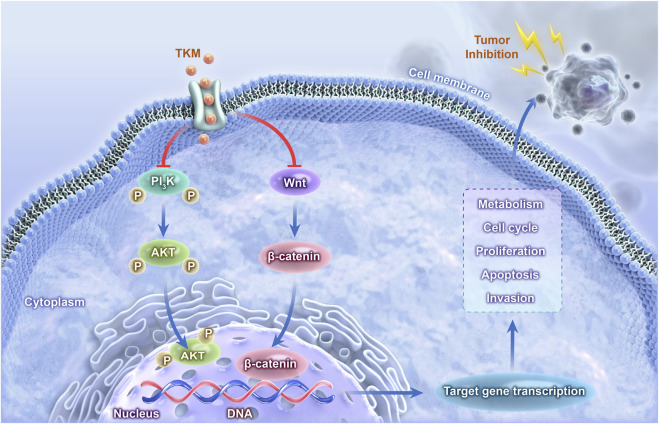
Molecular mechanism diagram.

This study used network pharmacology to predict multi-target mechanisms by which TKM may act against TNBC, and these proposed mechanisms are summarized in Figure 8. The antitumor effects of TKM in TNBC were supported by modulation of apoptosis, cell-cycle signaling, and cellular metabolic processes. This work provides a mechanistic framework for how TKM may function in TNBC; however, because several key targets and pathways remain prediction-based, our results should be interpreted as hypothesis-generating. Taken together, these findings suggest that TKM shows potential as a candidate for further preclinical development in TNBC rather than as a drug ready for clinical application.

## Data Availability

The data presented in the study are deposited in the MetaboLights repository, accession number MTBLS13902.
