# Supplementary material for: Integrative analysis of Trichosanthes kirilowii maxim formula granules’ anti-triple-negative breast cancer mechanism via network pharmacology, metabolomics, and molecular pharmacology
Source: Front Pharmacol. 2026 Mar 16;17:1657396. doi: 10.3389/fphar.2026.1657396 (PMC13033748; doi:10.3389/fphar.2026.1657396)
Supplement: Supplementary file 1 [file Supplementaryfile1.docx]

**Supplementary Methods**

**Qualitative HPLC–Orbitrap–MS/MS analysis of TKM granules**

For qualitative chemical profiling, 0.10 g of TKM formula granules was combined with 1,000 μL of 80% methanol (v/v in water). The mixture was ground for 5 min and vortexed for 10 min. The suspension was then centrifuged at 20,000 × g for 10 min at 4 °C, and the resulting supernatant was filtered through a membrane filter prior to LC–MS/MS analysis.

Chromatographic separation was performed on a Thermo Scientific UPLC system (Thermo Scientific, Germany) equipped with an AQ-C18 column. The mobile phases consisted of 0.01% formic acid in water (A) and methanol (B). The linear gradient elution program was as follows: 98% A and 2% B at 0 min, 80% B at 15 min, and 95% B at 27 min, which was maintained until 30 min. The flow rate was 0.30 mL/min, and the column temperature was kept constant (as specified by the manufacturer).

High-resolution MS detection and analysis were performed using a Q Exactive Orbitrap mass spectrometer (Thermo Fisher, USA) equipped with an electrospray ionization (ESI) source. The mass spectrometer was operated in data-dependent MS/MS mode with a full scan range from m/z 100.0 to 1,500.0 Da, continuously alternating between positive and negative ionization modes. The resolution was set to 70,000 for full MS scans and 17,500 for dd-MS^2 scans. The capillary temperature was 300 °C, the spray voltage was 3.2 kV, and the sheath gas flow rate was 40 arbitrary units. Data acquisition and processing were performed using Compound Discoverer 2.1 software (Thermo Fisher Scientific).

**Quantification of schisandrin in TKM granules by HPLC–UV**

The content of schisandrin in the TKM formula granules was determined by HPLC with UV detection using an external calibration curve constructed from a schisandrin reference standard. Briefly, an accurately weighed portion of TKM granules (0.10 g) was extracted with 1.0 mL of methanol–water (65:35, v/v) by vortexing and centrifugation as described above, and the supernatant was filtered through a 0.22 μm membrane filter prior to analysis.

Chromatographic separation was performed on a reversed-phase C18 column using methanol–water (65:35, v/v) as the mobile phase at a flow rate of 1.0 mL/min. The column temperature was maintained at 25 °C, the injection volume was 10 μL, and UV detection was carried out at 250 nm. Calibration solutions of schisandrin were prepared from a methanolic stock solution and diluted to a series of working concentrations covering the expected range in the TKM samples. The calibration curve was generated by plotting peak area versus nominal concentration, and the schisandrin content in TKM granules was calculated from the regression equation and expressed as mass of schisandrin per gram of granules. The final quantitative results are summarized in Supplementary Results and Table S3.

**Supplementary Results**

**Standard Curve**

A calibration curve for schisandrin was constructed using the external standard method. The relationship between concentration (x, mg/L) and peak area (y) was linear over the range of 0.5–150 mg/L, described by the equation:

y = 21,692.38 x + 7,658.57 (R = 0.99996, R² = 0.99991). The residual sum of squares (RSS) was 7.06 × 10⁷. The average response factor (RF) was 2.54 × 10⁻⁴, with a relative standard deviation (RSD) of 22.70%. Calibration data are summarized in Table S3.

**Quantitative Analysis**

The concentration of schisandrin in the sample was determined by interpolation from the calibration curve. The analyte exhibited a retention time of 5.581 min, with a peak area of 24,196. Based on the regression equation, the calculated concentration was **0.762 mg/L**. The content of schisandrin in the granules was calculated as **7.62 μg** per gram of dry weight, derived from the HPLC-determined concentration (0.762 mg/L) and the sample preparation protocol (0.1 g granules extracted and diluted to 1 mL).

**
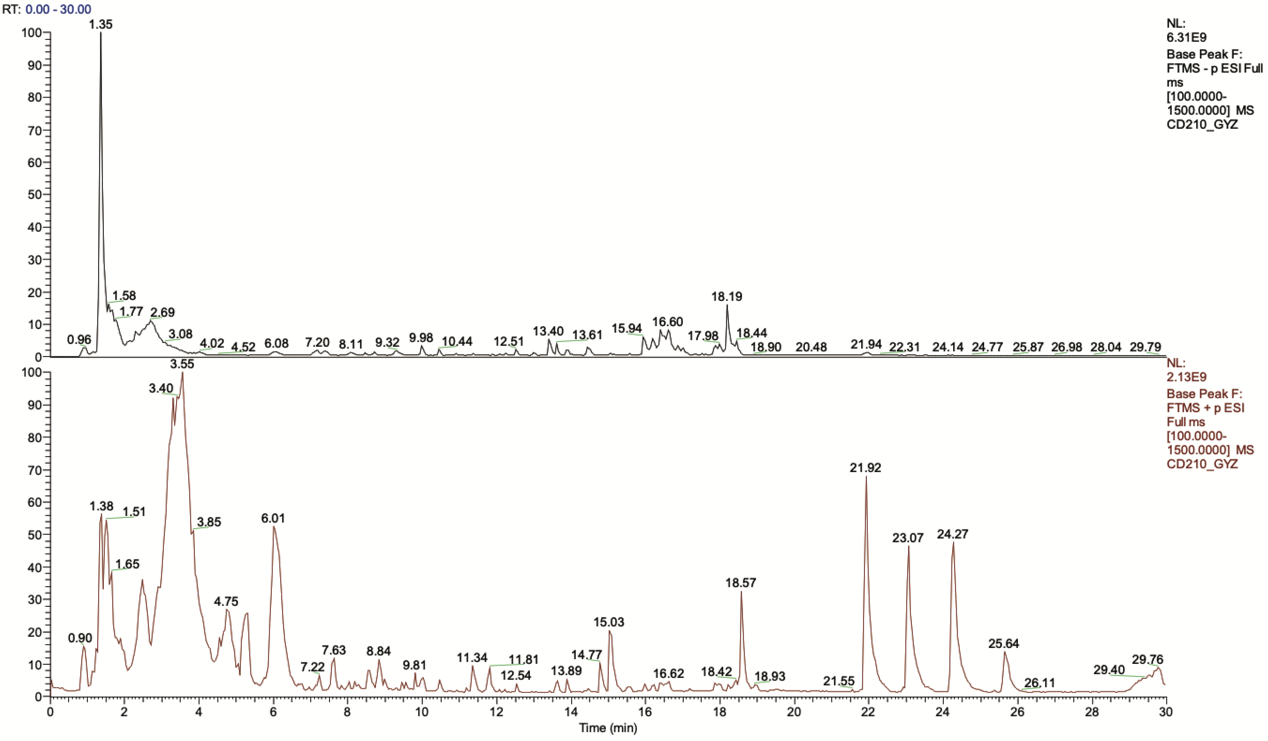
**

**Figure S1** Total ion chromatograms (TICs) of TKM in the negative ion mode (top) and positive ion mode (bottom).

**
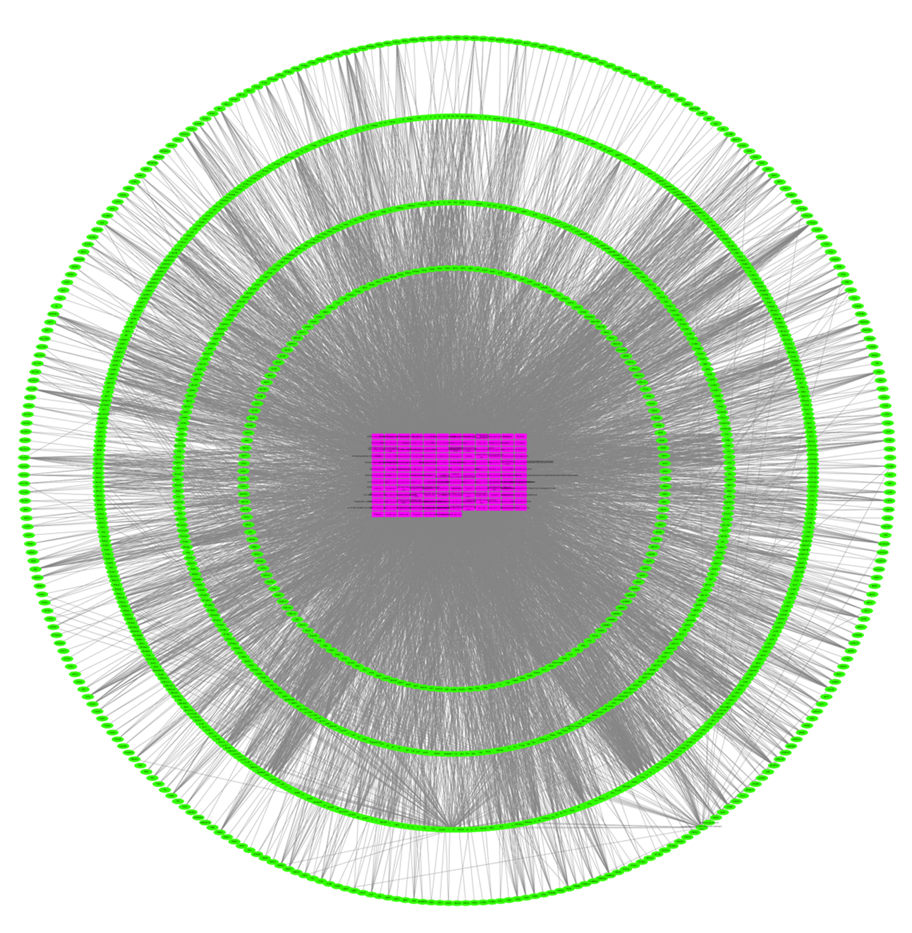
**

**Figure S2** Compound-target network of TKM. The network comprises 151 active ingredients of TKM and 1,164 putative targets. Red rhombuses represent chemical compounds and green circles represent targets.

**
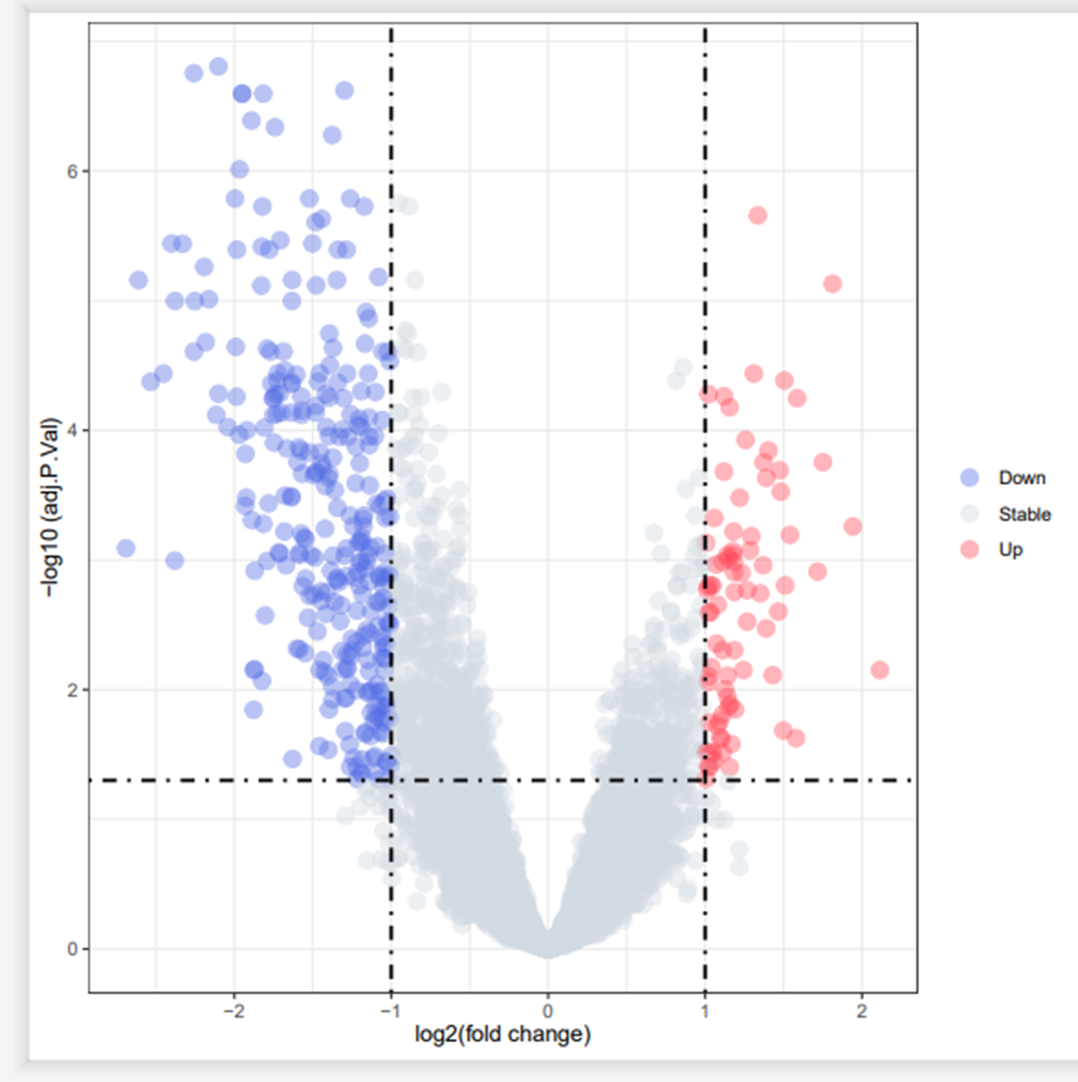
**

**Figure S3** Volcano plot of differential targets associated with breast cancer.

**
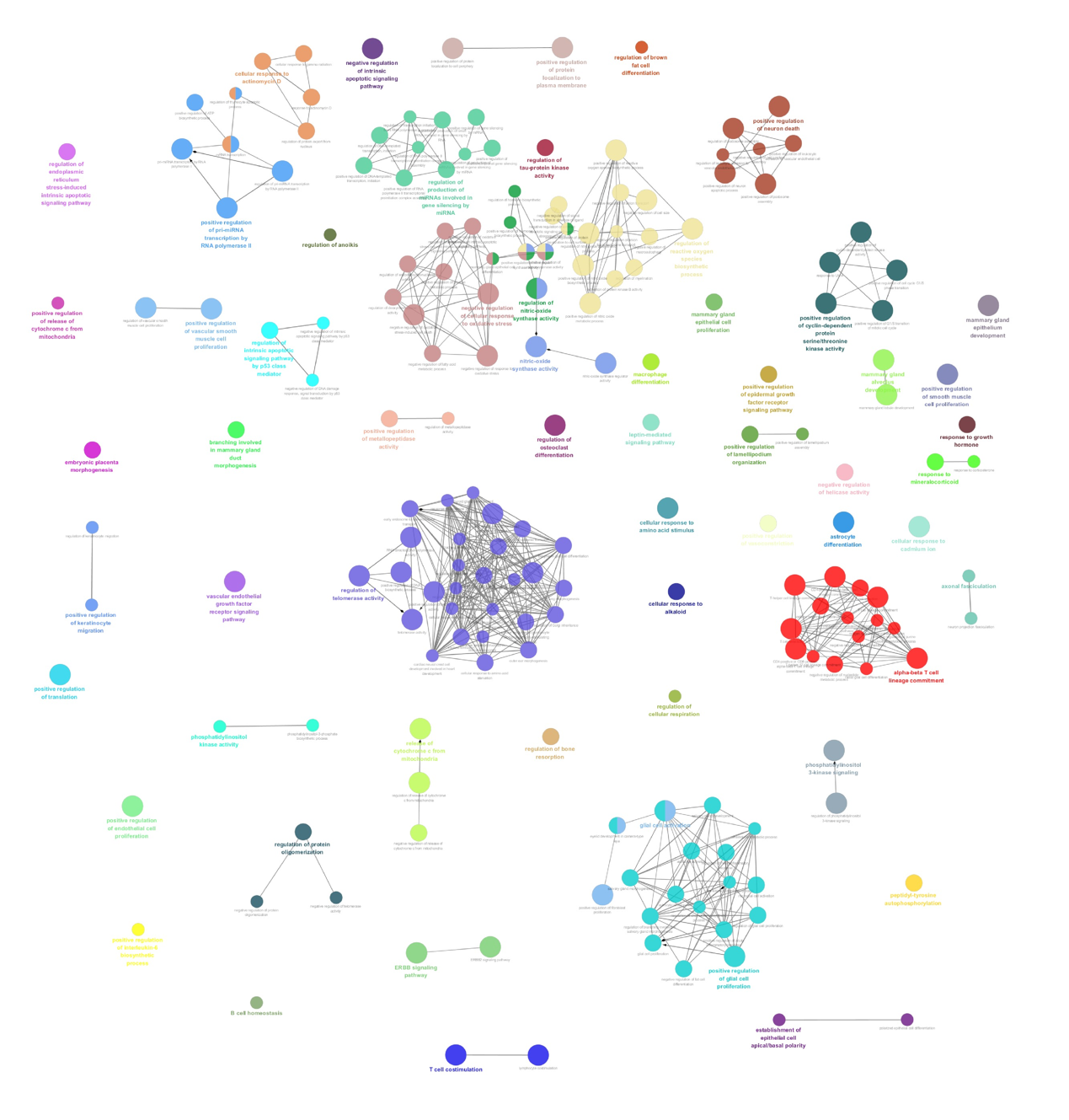
**

**Figure S4** ClueGO-based functional enrichment network of 214 targets. The network illustrates enriched Gene Ontology terms and pathways associated with the 214 targets. Each node represents an enriched biological process or pathway, while edges indicate shared genes between terms based on kappa score analysis. Different colors represent functionally related clusters. The analysis was performed using the ClueGO plugin in Cytoscape.

**
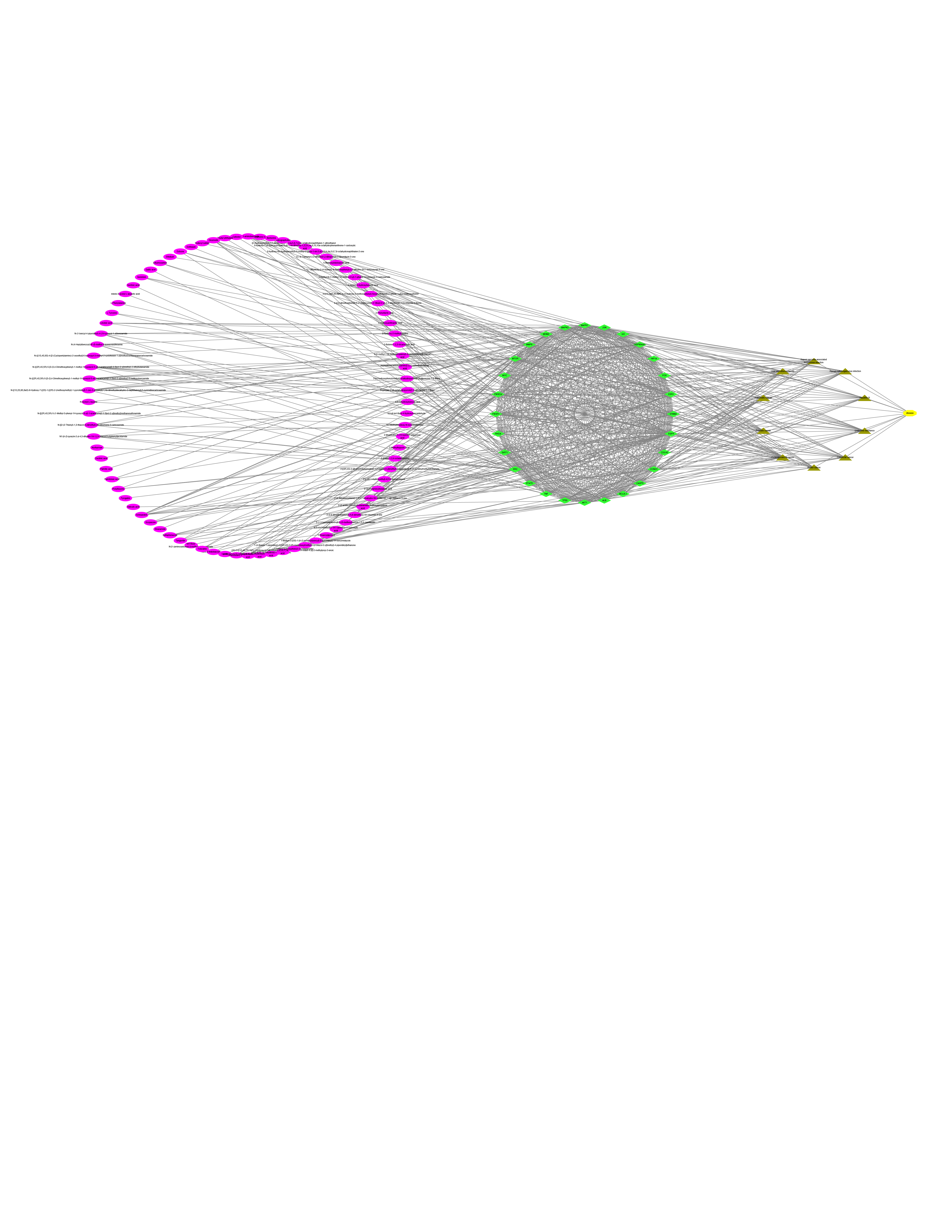
**

**Figure S5** Compound–target–pathway–disease network diagram.

**
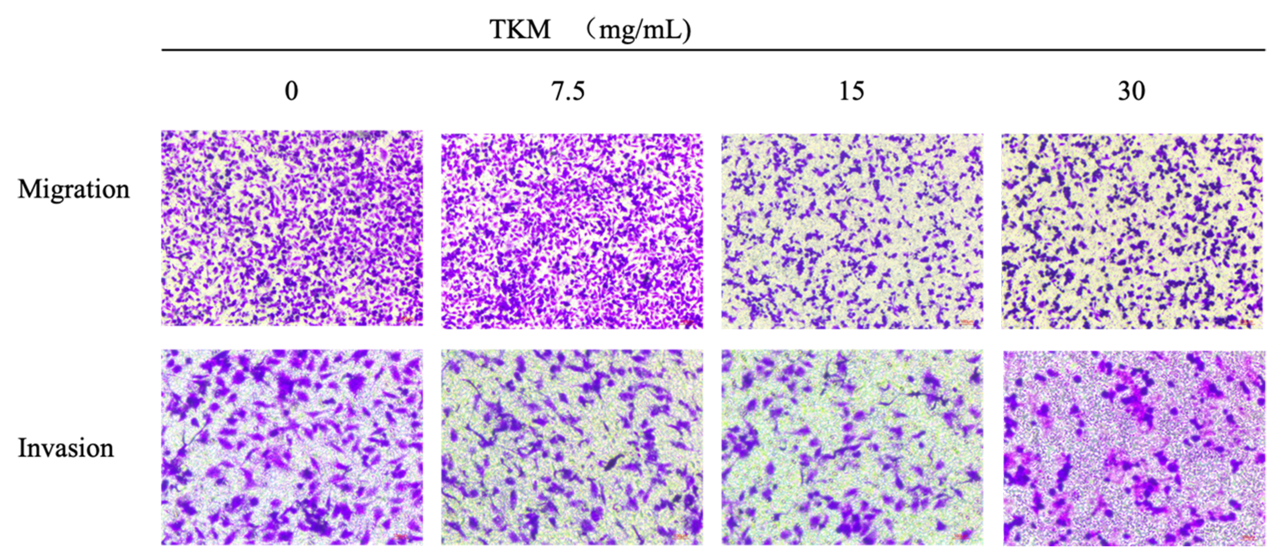
**

**Figure S6** Effects of TKM on migration and invasion in MDA-MB-231 cells.

**
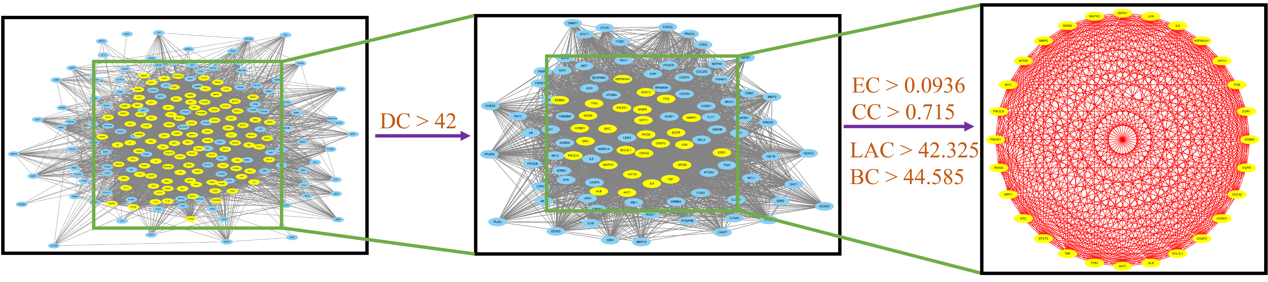
**

**Figure S7** The original high-resolution image for Figure2 (B)

**Table S1** Identification of 293 compounds detected in TKM granules

| **Name** | **Formula** | **mzCloud Best Match** |
| --- | --- | --- |
| Linoleic acid | C18 H32 O2 | 99.9 |
| Adenosine | C10 H13 N5 O4 | 99.9 |
| L-Phenylalanine | C9 H11 N O2 | 99.9 |
| 2,3,4,9-Tetrahydro-1H-β-carboline-3-carboxylic acid | C12 H12 N2 O2 | 99.9 |
| L-Isoleucine | C6 H13 N O2 | 99.8 |
| Oleanolic acid | C30 H48 O3 | 99.8 |
| Dibutyl phthalate | C16 H22 O4 | 99.8 |
| Benzoic acid | C7 H6 O2 | 99.8 |
| Salicylic acid | C7 H6 O3 | 99.7 |
| 16-Hydroxyhexadecanoic acid | C16 H32 O3 | 99.7 |
| Trigonelline | C7 H7 N O2 | 99.6 |
| Bis(2-ethylhexyl) phthalate | C24 H38 O4 | 99.6 |
| Dipropyleneglycol dibenzoate | C20 H22 O5 | 99.5 |
| Docosanamide | C22 H45 N O | 99.5 |
| DL-Stachydrine | C7 H13 N O2 | 99.5 |
| 4-Oxoproline | C5 H7 N O3 | 99.5 |
| 2-(Acetylamino)hexanoic acid | C8 H15 N O3 | 99.5 |
| 4-Oxoproline | C5 H7 N O3 | 99.5 |
| Salicylic acid | C7 H6 O3 | 99.5 |
| Azelaic acid | C9 H16 O4 | 99.5 |
| Nicotinic acid | C6 H5 N O2 | 99.5 |
| Norharman | C11 H8 N2 | 99.5 |
| Diosmetin | C16 H12 O6 | 99.4 |
| Bis(4-ethylbenzylidene)sorbitol | C24 H30 O6 | 99.4 |
| Bis(4-ethylbenzylidene)sorbitol | C24 H30 O6 | 99.4 |
| L-Norleucine | C6 H13 N O2 | 99.4 |
| (11E,15Z)-9,10,13-trihydroxyoctadeca-11,15-dienoic acid | C18 H32 O5 | 99.3 |
| D-Glucosamine | C6 H13 N O5 | 99.3 |
| Citroflex 4 | C18 H32 O7 | 99.3 |
| (11E,15Z)-9,10,13-trihydroxyoctadeca-11,15-dienoic acid | C18 H32 O5 | 99.3 |
| (11E,15Z)-9,10,13-trihydroxyoctadeca-11,15-dienoic acid | C18 H32 O5 | 99.3 |
| Gentisic acid | C7 H6 O4 | 99.3 |
| Sphingosine (d18:1) | C18 H37 N O2 | 99.2 |
| Palmitic acid | C16 H32 O2 | 99.2 |
| Guanine | C5 H5 N5 O | 99.2 |
| Bis(2-ethylhexyl)adipate | C22 H42 O4 | 99.2 |
| 3-Hydroxypicolinic acid | C6 H5 N O3 | 99.2 |
| (12Z)-9,10,11-trihydroxyoctadec-12-enoic acid | C18 H34 O5 | 99.1 |
| Oleic acid alkyne | C18 H30 O2 | 99.1 |
| 2-Hydroxyhippuric acid | C9 H9 N O4 | 99 |
| Isophthalic acid | C8 H6 O4 | 99 |
| (12Z)-9,10,11-trihydroxyoctadec-12-enoic acid | C18 H34 O5 | 99 |
| 4-Indolecarbaldehyde | C9 H7 N O | 99 |
| Methyl palmitate | C17 H34 O2 | 98.9 |
| Citraconic acid | C5 H6 O4 | 98.9 |
| Citric acid | C6 H8 O7 | 98.9 |
| 2-(3,4-Dihydroxyphenyl)-5-hydroxy-4-oxo-4H-chromen-7-yl 6-O-(6-deoxy-alpha-L-mannopyranosyl)-beta-D-glucopyranoside | C27 H30 O15 | 98.9 |
| L-Tyrosine | C9 H11 N O3 | 98.9 |
| Tangeritin | C20 H20 O7 | 98.9 |
| 2-(Acetylamino)hexanoic acid | C8 H15 N O3 | 98.8 |
| 3-(propan-2-yl)-octahydropyrrolo[1,2-a]pyrazine-1,4-dione | C10 H16 N2 O2 | 98.8 |
| 5-Hydroxymethyl-2-furaldehyde | C6 H6 O3 | 98.7 |
| 2-(2-amino-3-methylbutanamido)-3-phenylpropanoic acid | C14 H20 N2 O3 | 98.6 |
| Betaine | C5 H11 N O2 | 98.6 |
| Stearamide | C18 H37 N O | 98.5 |
| Dipentyl phthalate | C18 H26 O4 | 98.4 |
| Bis(3,5,5-trimethylhexyl) phthalate | C26 H42 O4 | 98.4 |
| 7-{[(2S,3R,4S,5S,6R)-4,5-dihydroxy-6-(hydroxymethyl)-3-{[(2S,3R,4R,5R,6S)-3,4,5-trihydroxy-6-methyloxan-2-yl]oxy}oxan-2-yl]oxy}-5-hydroxy-2-(4-hydroxy-3-methoxyphenyl)-4H-chromen-4-one | C28 H32 O15 | 98.4 |
| 9-Oxo-10(E),12(E)-octadecadienoic acid | C18 H30 O3 | 98.4 |
| Corchorifatty acid F | C18 H32 O5 | 98.4 |
| Choline | C5 H13 N O | 98.3 |
| Schisandrin | C24 H32 O7 | 98.3 |
| Oxolinic acid | C13 H11 N O5 | 98.3 |
| Methyl palmitate | C17 H34 O2 | 98.2 |
| 6-Hydroxycaproic acid | C6 H12 O3 | 98.2 |
| Oleic acid | C18 H34 O2 | 98.1 |
| Didecyl phthalate | C28 H46 O4 | 98.1 |
| Adenine | C5 H5 N5 | 97.9 |
| Hypoxanthine | C5 H4 N4 O | 97.8 |
| Stearic acid | C18 H36 O2 | 97.7 |
| Cynaroside | C21 H20 O11 | 97.7 |
| (4S)-4-hydroxy-3,5,5-trimethyl-4-[(1E)-3-{[(2R,3R,4S,5S,6R)-3,4,5-trihydroxy-6-(hydroxymethyl)oxan-2-yl]oxy}but-1-en-1-yl]cyclohex-2-en-1-one | C19 H30 O8 | 97.6 |
| Berberine | C20 H17 N O4 | 97.6 |
| 1,7-Bis(4-hydroxyphenyl)-3,5-heptanediol | C19 H24 O4 | 97.5 |
| 6-Hydroxycaproic acid | C6 H12 O3 | 97.5 |
| Suberic acid | C8 H14 O4 | 97.5 |
| Palmitoyl ethanolamide | C18 H37 N O2 | 97.4 |
| 3-[(4-hydroxyphenyl)methyl]-octahydropyrrolo[1,2-a]pyrazine-1,4-dione | C14 H16 N2 O3 | 97.3 |
| 3-hydroxy-4-(3-hydroxyphenyl)-1,2-dihydroquinolin-2-one | C15 H11 N O3 | 97.2 |
| Nobiletin | C21 H22 O8 | 97.1 |
| 5-hydroxy-2-(4-hydroxyphenyl)-6-methoxy-7-{[(2S,3R,4S,5S,6R)-3,4,5-trihydroxy-6-(hydroxymethyl)oxan-2-yl]oxy}-4H-chromen-4-one | C22 H22 O11 | 97 |
| 5,7-dihydroxy-2-(4-hydroxy-3-methoxyphenyl)-3,4-dihydro-2H-1-benzopyran-4-one | C16 H14 O6 | 96.9 |
| Neochlorogenic acid | C16 H18 O9 | 96.9 |
| D-(+)-Pyroglutamic Acid | C5 H7 N O3 | 96.8 |
| 1-Stearoylglycerol | C21 H42 O4 | 96.7 |
| (4S)-4-hydroxy-3,5,5-trimethyl-4-[(1E)-3-{[(2R,3R,4S,5S,6R)-3,4,5-trihydroxy-6-(hydroxymethyl)oxan-2-yl]oxy}but-1-en-1-yl]cyclohex-2-en-1-one | C19 H30 O8 | 96.7 |
| Pyrogallol | C6 H6 O3 | 96.7 |
| L-Phenylalanine | C9 H11 N O2 | 96.5 |
| Hexadecanamide | C16 H33 N O | 96.5 |
| Dodecyl sulfate | C12 H26 O4 S | 96.2 |
| Methylsuccinic acid | C5 H8 O4 | 96.1 |
| 2-[(1S)-1-Hydroxyethyl]-4(1H)-quinazolinone | C10 H10 N2 O2 | 96 |
| L-Iditol | C6 H14 O6 | 96 |
| Thymidine | C10 H14 N2 O5 | 96 |
| 2'-O-Methyladenosine | C11 H15 N5 O4 | 96 |
| Tolycaine | C15 H22 N2 O3 | 96 |
| 3-(1-hydroxyethyl)-2,3,6,7,8,8a-hexahydropyrrolo[1,2-a]pyrazine-1,4-dione | C9 H14 N2 O3 | 95.8 |
| Corchorifatty acid F | C18 H32 O5 | 95.7 |
| D-(+)-Pyroglutamic Acid | C5 H7 N O3 | 95.7 |
| N-Acetyl-L-leucine | C8 H15 N O3 | 95.7 |
| 4-methyl-2-oxo-1,2-dihydroquinoline-3-carbonitrile | C11 H8 N2 O | 95.6 |
| Cyclo(phenylalanyl-prolyl) | C14 H16 N2 O2 | 95.5 |
| 2-Phenylethyl 3-O-(4-carboxy-3-hydroxy-3-methylbutanoyl)-β-D-glucopyranoside | C20 H28 O10 | 95.4 |
| octadec-9-ynoic acid | C18 H32 O2 | 95.3 |
| 3,5-di-tert-Butyl-4-hydroxybenzaldehyde | C15 H22 O2 | 95.3 |
| (+/-)12(13)-DiHOME | C18 H34 O4 | 95.2 |
| 2-(3,4-Dihydroxyphenyl)ethyl 3-O-(6-deoxy-β-L-mannopyranosyl)-6-O-[(2E)-3-(3,4-dihydroxyphenyl)-2-propenoyl]-β-D-glucopyranoside | C29 H36 O15 | 95.2 |
| 12-Oxo phytodienoic acid | C18 H28 O3 | 95 |
| 12-Oxo phytodienoic acid | C18 H28 O3 | 95 |
| (+/-)12(13)-DiHOME | C18 H34 O4 | 94.9 |
| Methylsuccinic acid | C5 H8 O4 | 94.8 |
| Corchorifatty acid F | C18 H32 O5 | 94.8 |
| α-Eleostearic acid | C18 H30 O2 | 94.7 |
| DL-Arginine | C6 H14 N4 O2 | 94.6 |
| Genistein | C15 H10 O5 | 94.6 |
| Tolycaine | C15 H22 N2 O3 | 94.4 |
| N-Acetyl-L-leucine | C8 H15 N O3 | 94.1 |
| 2-(3,4-Dihydroxyphenyl)ethyl 3-O-(6-deoxy-β-L-mannopyranosyl)-6-O-[(2E)-3-(3,4-dihydroxyphenyl)-2-propenoyl]-β-D-glucopyranoside | C29 H36 O15 | 94.1 |
| Methyl palmitate | C17 H34 O2 | 93.8 |
| (2R)-7-Methoxy-3-oxo-3,4-dihydro-2H-1,4-benzoxazin-2-yl β-D-glucopyranoside | C15 H19 N O9 | 93.8 |
| D-(+)-Pipecolinic acid | C6 H11 N O2 | 93.8 |
| trans-3-Indoleacrylic acid | C11 H9 N O2 | 93.7 |
| 16-Hydroxyhexadecanoic acid | C16 H32 O3 | 93.7 |
| 12-Oxo phytodienoic acid | C18 H28 O3 | 93.7 |
| DL-β-Leucine | C6 H13 N O2 | 93.6 |
| 3-Hydroxyanthranilic acid | C7 H7 N O3 | 93.6 |
| 1,2,3-cyclopropanetricarboxylic acid | C6 H6 O6 | 93.6 |
| Gentisic acid | C7 H6 O4 | 93.6 |
| Tridemorph | C19 H39 N O | 93.4 |
| 3-Amino-2-naphthoic acid | C11 H9 N O2 | 93.4 |
| 1-Linoleoyl glycerol | C21 H38 O4 | 93.3 |
| 1-Linoleoyl glycerol | C21 H38 O4 | 93.3 |
| 4-oxododecanedioic acid | C12 H20 O5 | 93 |
| 3-Amino-2-naphthoic acid | C11 H9 N O2 | 93 |
| 2,3-dihydroxypropyl 12-methyltridecanoate | C17 H34 O4 | 92.9 |
| 4-Acetamidobutanoic acid | C6 H11 N O3 | 92.9 |
| N-Acetylvaline | C7 H13 N O3 | 92.5 |
| 1,2,3-cyclopropanetricarboxylic acid | C6 H6 O6 | 92.4 |
| α-Eleostearic acid | C18 H30 O2 | 92.3 |
| (±)-Abscisic acid | C15 H20 O4 | 92.1 |
| N-Acetylvaline | C7 H13 N O3 | 91.6 |
| Dodecanedioic acid | C12 H22 O4 | 91.4 |
| 4,5-Dicaffeoylquinic acid | C25 H24 O12 | 91 |
| D-Glucose 6-phosphate | C6 H13 O9 P | 90.8 |
| 3-Hydroxy-2-methylpyridine | C6 H7 N O | 90.8 |
| Prolylleucine | C11 H20 N2 O3 | 90.7 |
| Pyrogallol | C6 H6 O3 | 90.5 |
| 4-(2,3-dihydro-1,4-benzodioxin-6-yl)-1,2-diphenylbut-2-ene-1,4-dione | C24 H18 O4 | 90.3 |
| (+/-)9-HpODE | C18 H32 O4 | 90.2 |
| Vanillin | C8 H8 O3 | 90.1 |
| 6-formyl-10-(hydroxymethyl)-5-methoxy-3-methylidene-2-oxo-2H,3H,3aH,4H,5H,8H,9H,11aH-cyclodeca[b]furan-4-yl 2-methylbutanoate | C21 H28 O7 | 90.1 |
| Cafestol | C20 H28 O3 | 90.1 |
| 6-formyl-10-(hydroxymethyl)-5-methoxy-3-methylidene-2-oxo-2H,3H,3aH,4H,5H,8H,9H,11aH-cyclodeca[b]furan-4-yl 2-methylbutanoate | C21 H28 O7 | 89.9 |
| 2-[(2S,3R,4S,5R)-3,4-Dihydroxy-5-{[(isopropylcarbamoyl)amino]methyl}tetrahydro-2-furanyl]-N-[2-(dimethylamino)ethyl]acetamide | C15 H30 N4 O5 | 89.7 |
| Caprolactam | C6 H11 N O | 89.4 |
| 4-Aminobenzoic acid | C7 H7 N O2 | 89.4 |
| Bis(3,5,5-trimethylhexyl) phthalate | C26 H42 O4 | 89.4 |
| 13(S)-HOTrE | C18 H30 O3 | 89.4 |
| 3-Phenyllactic acid | C9 H10 O3 | 89.4 |
| Isophthalic acid | C8 H6 O4 | 89.3 |
| 13(S)-HOTrE | C18 H30 O3 | 89.2 |
| tert-Butyl N-[1-(aminocarbonyl)-3-methylbutyl]carbamate | C11 H22 N2 O3 | 89 |
| Thymidine | C10 H14 N2 O5 | 88.8 |
| (15Z)-9,12,13-Trihydroxy-15-octadecenoic acid | C18 H34 O5 | 88.7 |
| Arjungenin | C30 H48 O6 | 88.5 |
| tert-Butyl N-[1-(aminocarbonyl)-3-methylbutyl]carbamate | C11 H22 N2 O3 | 88.4 |
| 4-Amino-3-hydroxybenzoic acid | C7 H7 N O3 | 88.3 |
| 11-dehydro Thromboxane B2 | C20 H32 O6 | 88 |
| α-Linolenic acid | C18 H30 O2 | 87.7 |
| Gallic acid | C7 H6 O5 | 87.6 |
| 6-formyl-10-(hydroxymethyl)-5-methoxy-3-methylidene-2-oxo-2H,3H,3aH,4H,5H,8H,9H,11aH-cyclodeca[b]furan-4-yl 2-methylbutanoate | C21 H28 O7 | 87.4 |
| Ageratriol | C15 H24 O3 | 87.2 |
| Hypoxanthine | C5 H4 N4 O | 87.1 |
| (15Z)-9,12,13-Trihydroxy-15-octadecenoic acid | C18 H34 O5 | 87 |
| (2E)-3-[(1R,4S,7R,7aR)-1-hydroxy-3,7-dimethyl-2,4,5,6,7,7a-hexahydro-1H-inden-4-yl]-2-methylprop-2-enoic acid | C15 H22 O3 | 86.8 |
| 3,5-Dihydroxybenzoic acid | C7 H6 O4 | 86.4 |
| Ageratriol | C15 H24 O3 | 86.3 |
| Prolylleucine | C11 H20 N2 O3 | 86.1 |
| Dihydromorphine | C17 H21 N O3 | 86 |
| 1-Linoleoyl glycerol | C21 H38 O4 | 85.9 |
| Butyl 4-aminobenzoate | C11 H15 N O2 | 85.6 |
| Prolylleucine | C11 H20 N2 O3 | 85.6 |
| Andrographolide | C20 H30 O5 | 85.5 |
| 6-Methylquinoline | C10 H9 N | 85.5 |
| 11-piperidino-2,3-dihydro-1H-cyclopenta[4,5]pyrido[1,2-a]benzimidazole-4-carbonitrile | C20 H20 N4 | 84.7 |
| L-Iditol | C6 H14 O6 | 84.5 |
| 4-(2,6-dimethylphenyl)-5-(7-oxabicyclo[2.2.1]hept-2-yl)-2,4-dihydro-3H-1,2,4-triazole-3-thione | C16 H19 N3 O S | 84.5 |
| Tetradecanedioic acid | C14 H26 O4 | 84.4 |
| Tangeritin | C20 H20 O7 | 84.3 |
| (1S,5R,9R,13R)-1,5,9-trimethyl-11,14,15,16-tetraoxatetracyclo[10.3.1.0â´,ÂaÂl.0â¸,ÂaÂl]hexadecan-10-one | C15 H22 O5 | 84 |
| 2-(1,3-benzodioxol-5-yl)-5-(3-methoxybenzyl)-1,3,4-oxadiazole | C17 H14 N2 O4 | 84 |
| 2-Aminooctanedioic acid | C8 H15 N O4 | 83.9 |
| N1,N1-dimethyl-3-chloro-4-(7,8-dimethyl-1,5-dihydro-2,4-benzodithiepin-3-yl)aniline | C19 H22 Cl N S2 | 83.8 |
| Octylphosphonic acid | C8 H19 O3 P | 83.3 |
| Tetradecanedioic acid | C14 H26 O4 | 83.1 |
| Pyridoxal | C8 H9 N O3 | 83 |
| 2-oxa-4-azatetracyclo[6.3.1.1~6,10~.0~1,5~]tridecan-3-one | C11 H15 N O2 | 82.8 |
| Uric acid | C5 H4 N4 O3 | 82.8 |
| Palmitic Acid | C16 H32 O2 | 82.8 |
| 2-[(2R,4aR,8R,8aR)-8-hydroxy-4a,8-dimethyl-decahydronaphthalen-2-yl]prop-2-enoic acid | C15 H24 O3 | 82.8 |
| Uridine | C9 H12 N2 O6 | 82.8 |
| 2-(2,6-dimethoxyphenyl)-5,6-dimethoxy-4H-chromen-4-one | C19 H18 O6 | 82.6 |
| 7-(1H-pyrrol-1-yl)-5H-chromeno[2,3-b]pyridin-5-one | C16 H10 N2 O2 | 82.4 |
| 9-hydroxy-2,10,10-trimethyltricyclo[6.3.0.0Âa,âµ]undec-6-ene-6-carboxylic acid | C15 H22 O3 | 82.4 |
| 3-tert-Butyladipic acid | C10 H18 O4 | 82.1 |
| N-({(2R,4S,5R)-5-[3-(3,4-Dimethoxyphenyl)-1-methyl-1H-pyrazol-5-yl]-1-azabicyclo[2.2.2]oct-2-yl}methyl)-4-methoxybenzamide | C28 H34 N4 O4 | 82 |
| 2-[(2S,3R,4S,5R)-3,4-Dihydroxy-5-{[(isopropylcarbamoyl)amino]methyl}tetrahydro-2-furanyl]-N-[2-(dimethylamino)ethyl]acetamide | C15 H30 N4 O5 | 82 |
| 4-[(1S,3aR,4S,6aR)-4-(4-hydroxy-3-methoxyphenyl)-hexahydrofuro[3,4-c]furan-1-yl]-2-methoxyphenol | C20 H22 O6 | 81.7 |
| 4-Methylumbelliferone hydrate | C10 H8 O3 | 81.5 |
| N-(2,4-Dimethylphenyl)formamide | C9 H11 N O | 81.4 |
| methyl 2-[4-ethenyl-2,6-dihydroxy-3-(3-hydroxyprop-1-en-2-yl)-4-methylcyclohexyl]prop-2-enoate | C16 H24 O5 | 81.1 |
| N-(1-benzyl-4-piperidinyl)-4-(1H-pyrazol-1-yl)benzamide | C22 H24 N4 O | 81.1 |
| alpha-D-Glucopyranosyl 2-O-(2-methylbutanoyl)-alpha-D-glucopyranoside | C17 H30 O12 | 80.9 |
| 4-(2,6-dimethylphenyl)-5-(7-oxabicyclo[2.2.1]hept-2-yl)-2,4-dihydro-3H-1,2,4-triazole-3-thione | C16 H19 N3 O S | 80.9 |
| (±)9-HpODE | C18 H32 O4 | 80.7 |
| 4-oxododecanedioic acid | C12 H20 O5 | 80.5 |
| N-({(2R,4S,5R)-5-[3-(3,4-Dimethoxyphenyl)-1-methyl-1H-pyrazol-5-yl]-1-azabicyclo[2.2.2]oct-2-yl}methyl)-2-ethylbutanamide | C26 H38 N4 O3 | 80.3 |
| L(-)-Carnitine | C7 H15 N O3 | 80.3 |
| Butyl 4-aminobenzoate | C11 H15 N O2 | 80.2 |
| Ethidium | C21 H19 N3 | 80 |
| 1,9b-Dihydroxy-6,6,9a-trimethyl-5,5a,6,7,8,9,9a,9b-octahydronaphtho[1,2-c]furan-3(1H)-one | C15 H22 O4 | 79.9 |
| Cuminaldehyde | C10 H12 O | 79.6 |
| trans,trans-Muconic acid | C6 H6 O4 | 79.6 |
| (1'S,2'S)-3',11'-dihydroxy-1',2',5'-trimethyl-8'-oxaspiro[oxirane-2,12'-tricyclo[7.2.1.0Â?,â·]dodecan]-5'-en-4'-one | C15 H20 O5 | 79.3 |
| N1-[4-(3-pyrazin-2-yl-4,5-dihydro-1H-1,2,4-triazol-5-yl)phenyl]acetamide | C14 H14 N6 O | 79.3 |
| (+/-)-C75 | C14 H22 O4 | 79.2 |
| N-tert-Butyl-α-phenylnitrone | C11 H15 N O | 79.1 |
| 3,4-Methylenedioxy-N-benzylcathinone | C17 H17 N O3 | 79 |
| 2-Isopropyl-5-thieno[3,2-b]thiophen-2-yl-1,3,4-oxadiazole | C11 H10 N2 O S2 | 78.9 |
| 2,2,6,6-Tetramethyl-1-piperidinol (TEMPO) | C9 H19 N O | 78 |
| 5-Methoxyindoleacetic acid | C11 H11 N O3 | 77.9 |
| 6-Hydroxypicolinic acid | C6 H5 N O3 | 77.8 |
| 10-Nitrolinoleate | C18 H31 N O4 | 77.5 |
| 2-Amino-1,3,4-octadecanetriol | C18 H39 N O3 | 77.5 |
| DL-Malic acid | C4 H6 O5 | 77.4 |
| Heptadecanoic Acid | C17 H34 O2 | 77.3 |
| Cathinone | C9 H11 N O | 77.2 |
| AB-CHMICA | C21 H29 N3 O2 | 77.2 |
| 6-Acetylmorphine | C19 H21 N O4 | 77.2 |
| Inosine | C10 H12 N4 O5 | 76.8 |
| Ethylmorphine | C19 H23 N O3 | 76.8 |
| 13,14-dihydro-15-keto-tetranor Prostaglandin F1? | C16 H28 O5 | 76.7 |
| Xanthosine | C10 H12 N4 O6 | 76.6 |
| 2-(4-Nitrobenzylidene)-2,3,6,7-tetrahydro-5H-imidazo[2,1-b][1,3]thiazin-3-one | C13 H11 N3 O3 S | 76.4 |
| 2-Methylhippuric acid | C10 H11 N O3 | 76.4 |
| 9-hydroxy-7-(2-hydroxypropan-2-yl)-1,4a-dimethyl-1,2,3,4,4a,9,10,10a-octahydrophenanthrene-1-carboxylic acid | C20 H28 O4 | 76.4 |
| Indole-3-acetyl-L-aspartic acid | C14 H14 N2 O5 | 76.2 |
| [2-(hydroxymethyl)-5,5,8a-trimethyl-1,4,4a,5,6,7,8,8a-octahydronaphthalen-1-yl]methanol | C15 H26 O2 | 75.8 |
| 2-(hydroxymethyl)-6-[(E)-4-(1,2,4-trihydroxy-2,6,6-trimethylcyclohexyl)but-3-en-2-yl]oxyoxane-3,4,5-triol | C19 H34 O9 | 75.8 |
| 3-Butene-1,2,3-tricarboxylic acid | C7 H8 O6 | 75.5 |
| Thymidine 5'-monophosphate | C10 H15 N2 O8 P | 75.3 |
| Rifampicin | C43 H58 N4 O12 | 75.1 |
| 3-[(methoxycarbonyl)amino]-2,2,3-trimethylbutanoic acid | C9 H17 N O4 | 74.8 |
| Diacetoxyscirpenol | C19 H26 O7 | 74.8 |
| 3-(2-Hydroxyethyl)indole | C10 H11 N O | 74.8 |
| (±)-Abscisic acid | C15 H20 O4 | 74.8 |
| Sorbic acid | C6 H8 O2 | 74.7 |
| N1-imino(2-methyl-1,3-thiazol-4-yl)methyl-4-(trifluoromethoxy)benzamide | C13 H10 F3 N3 O2 S | 74.6 |
| 3-Hydroxy-2-methylpyridine | C6 H7 N O | 74.5 |
| n-Pentyl isopentyl phthalate | C18 H26 O4 | 74.4 |
| 3-[(methoxycarbonyl)amino]-2,2,3-trimethylbutanoic acid | C9 H17 N O4 | 74.3 |
| Sulfaphenazole | C15 H14 N4 O2 S | 74.3 |
| N-({(1S,4S,6S)-4-[2-(Cyclopentylamino)-2-oxoethyl]-6-isopropyl-3-methyl-2-cyclohexen-1-yl}methyl)cyclopropanecarboxamide | C22 H36 N2 O2 | 74.2 |
| AB-CHMICA | C21 H29 N3 O2 | 74.1 |
| N-[(1S,2S,8S,8aS)-8-Hydroxy-7-{(2S)-1-[(2R)-2-(methoxymethyl)-1-pyrrolidinyl]-1-oxo-2-propanyl}-1,4a-dimethyldecahydro-2-naphthalenyl]-5-pyrimidinecarboxamide | C26 H40 N4 O4 | 74 |
| Dothiepin | C19 H21 N S | 73.9 |
| 5-hydroxy-4-methoxy-5,6-dihydro-2H-pyran-2-one | C6 H8 O4 | 73.9 |
| 6,7,8-Triphenyl-2,3-dihydro[1,2,4]triazolo[4,3-b]pyridazin-3-one | C23 H16 N4 O | 73.6 |
| 2-Morpholino-5-(1H-pyrrol-1-yl)benzoic acid | C15 H16 N2 O3 | 73.6 |
| Sepiapterin | C9 H11 N5 O3 | 73.5 |
| N-Acetyl-DL-norvaline | C7 H13 N O3 | 73.1 |
| N-(4-Heptylbenzoyl)-N'-(5-methyl-3-isoxazolyl)thiourea | C19 H25 N3 O2 S | 72.8 |
| Scoparone | C11 H10 O4 | 72.8 |
| Estrone | C18 H22 O2 | 72.5 |
| 5-hydroxy-4-methoxy-5,6-dihydro-2H-pyran-2-one | C6 H8 O4 | 72.5 |
| 3-(2-Hydroxyethyl)indole | C10 H11 N O | 72.2 |
| Palmitoleic Acid | C16 H30 O2 | 72.1 |
| N-Acetyl-DL-norvaline | C7 H13 N O3 | 72.1 |
| L-Tyrosine | C9 H11 N O3 | 72 |
| 2-Isopropylmalic acid | C7 H12 O5 | 72 |
| 4-methyl-N-(1-methyl-1H-indazol-3-yl)-1-phenyl-1H-pyrazole-3-carboxamide | C19 H17 N5 O | 71.6 |
| 2-[(3S)-1-(Benzylsulfonyl)-3-pyrrolidinyl]-1-methyl-1H-benzimidazole | C19 H21 N3 O2 S | 71.4 |
| 3-[4-methyl-1-(2-methylpropanoyl)-3-oxocyclohexyl]butanoic acid | C15 H24 O4 | 71.2 |
| Ethylmorphine | C19 H23 N O3 | 71.1 |
| 6-hydroxy-4a-(hydroxymethyl)-5-methyl-3-(prop-1-en-2-yl)-2,3,4,4a,5,6,7,8-octahydronaphthalen-2-one | C15 H22 O3 | 71 |
| 1-Methyl-2-{(3S)-1-[4-(2-pyridinyl)benzyl]-3-pyrrolidinyl}-1H-benzimidazole | C24 H24 N4 | 70.7 |
| N-{[(2R,4S,5R)-5-(1-Methyl-3-phenyl-1H-pyrazol-5-yl)-1-azabicyclo[2.2.2]oct-2-yl]methyl}methanesulfonamide | C19 H26 N4 O2 S | 70.7 |
| 1-(4-Benzyl-1-piperidinyl)-2-[(3R,4S)-3-{[5-(cyclohexylmethyl)-1,2-oxazol-3-yl]methyl}-4-piperidinyl]ethanone | C30 H43 N3 O2 | 70.5 |
| Neostigmine | C12 H18 N2 O2 | 70.5 |
| 2-[(3R,4S)-3-{[5-(Cyclohexylmethyl)-1,2-oxazol-3-yl]methyl}-4-piperidinyl]-N-(3-pyridinylmethyl)acetamide | C24 H34 N4 O2 | 70.5 |
| 3-Hydroxy-2-methylpyridine | C6 H7 N O | 70.4 |
| Bisphenol A | C15 H16 O2 | 70 |
| N-{[2-(2-Thienyl)-1,3-thiazol-4-yl]methyl}-2H-chromene-3-carboxamide | C18 H14 N2 O2 S2 | 70 |

**Table S2** The Degree values of related components

**Table S3** Calibration data for schisandrin

| **Concentration (mg/L)** | **Mean Peak Area** |
| --- | --- |
| 0.5 | 17988 |
| 1 | 29216 |
| 10 | 222114 |
| 25 | 550619 |
| 75 | 1641457 |
| 150 | 3258187 |

**Table S4** Differential metabolites in negative ion mode

| **ID** | **Name** | **VIP** | **p-value** |
| --- | --- | --- | --- |
| M386T34 | 4-hydroxytamoxifen | 2.731611791 | 0.00150339 |
| M673T35_1 | 1-Hexadecanoyl-2-(9Z-octadecenoyl)-sn-glycero-3-phosphoric acid | 1.091128728 | 0.002028203 |
| M93T24 | Phenol | 3.296855572 | 0.00370722 |
| M103T224 | Beta-hydroxybutyrate | 2.195323386 | 0.005234133 |
| M771T143 | 1,2-dioleoyl-sn-glycero-3-phosphoethanolamine-n,n-dimethyl | 2.240035985 | 0.005867095 |
| M243T233 | Pseudouridine | 2.954795106 | 0.007946133 |
| M717T146 | 1-palmitoyl-3-oleoyl-sn-glycero-2-phosphoethanolamine | 3.913046058 | 0.012896797 |
| M153T233_1 | L-Asparagine | 1.268916915 | 0.014487814 |
| M719T94 | Pg 32:1 | 1.255902039 | 0.019566824 |
| M746T34 | 1-palmitoyl-2-linoleoyl-sn-glycero-3-phospho-(1'-rac-glycerol) | 3.9492427 | 0.023378221 |
| M438T197 | N-arachidonoyldopamine | 3.124454576 | 0.044026596 |
| M699T118 | 1-stearoyl-2-linoleoyl-sn-glycero-3-phosphate | 1.123570666 | 0.045315437 |
| M203T253 | DL-tryptophan | 1.697579108 | 0.047628929 |
| M715T147 | 2-linoleoyl-1-palmitoyl-sn-glycero-3-phosphoethanolamine | 1.428071766 | 0.048034698 |
| M173T426 | Cis-aconitate | 2.071216534 | 0.042225283 |

**Table S5** Differential metabolites in positive ion mode

| **ID** | **Name** | **VIP** | **p-value** |
| --- | --- | --- | --- |
| M149T255 | Stylopine | 1.639729341 | 0.002130611 |
| M173T320_2 | L-Isoleucine | 1.213509523 | 0.002347825 |
| M146T348 | 4-Guanidinobutyric acid | 1.801833092 | 0.002723869 |
| M138T277 | Trigonelline | 6.650662552 | 0.004902987 |
| M204T296 | Acetylcarnitine | 2.89722912 | 0.005934701 |
| M160T220 | Valine betaine | 1.662692989 | 0.008340989 |
| M118T264_2 | Betaine | 16.1114664 | 0.010062773 |
| M550T153 | 1-o-hexadecyl-2-o-(2e-butenoyl)-sn-glyceryl-3-phosphocholine | 1.995475468 | 0.011201106 |
| M331T443 | Ser-Pro-Lys | 1.246565245 | 0.011896198 |
| M72T289 | 1,2-diamino-2-methylpropane | 1.764631815 | 0.013104395 |
| M275T446 | Lys-Gln | 1.054489954 | 0.014032775 |
| M167T263 | Phenyllactic acid | 1.67200002 | 0.01648681 |
| M325T465 | Uridine 5'-monophosphate (UMP) | 1.297792847 | 0.018146459 |
| M146T366_2 | Deoxycarnitine | 8.479204418 | 0.02135785 |
| M508T189_3 | 1-(1z-octadecenyl)-sn-glycero-3-phosphocholine | 5.755108681 | 0.02222485 |
| M430T29 | (+)-.alpha.-tocopherol | 3.765696616 | 0.025204335 |
| M146T255 | DL-O-tyrosine | 1.375035787 | 0.02843542 |
| M120T352 | DL-threonine | 2.833769259 | 0.030509568 |
| M166T255 | DL-phenylalanine | 5.99577364 | 0.031863142 |
| M291T473 | Argininosuccinic acid | 6.201045998 | 0.037038226 |
| M642T28_1 | 1-linoleoyl-2-oleoyl-rac-glycerol | 2.500318272 | 0.037415747 |
| M424T168 | Linoleoylcarnitine | 2.6961315 | 0.040544899 |
| M175T569 | L-Arginine | 7.214204973 | 0.040707693 |
| M248T311_1 | 3-hydroxybutyrylcarnitine | 9.980847373 | 0.040790264 |
| M301T477 | Gly-Pro-Lys | 1.271339531 | 0.044804269 |
| M86T261 | 1,5-pentanediamine | 4.280584113 | 0.047211679 |
| M150T93 | Pyridoxal | 2.353368533 | 0.047292282 |
| M102T44 | N-(.beta.-ketocaproyl)-dl-homoserine lactone | 1.272347022 | 0.048216453 |

**Table S6** Differential amino acids and related metabolites in TKM-treated 4T1 cells

| **Metabolite name** | **KEGG ID** | **HMDB ID** | **Retention time (min)** | **Fold change (TKM / control)** | **p value** |
| --- | --- | --- | --- | --- | --- |
| Phosphoserine | C01005 | HMDB0000272 | 8.947517 | 0.358492002 | 8.38E-06 |
| Cis-4-Hydroxy-D-proline | C03440 | HMDB0060460 | 6.184200333 | 0.376846918 | 0.000261 |
| 1-Methylhistidine | C01152 | HMDB0000001 | 6.184200333 | 0.41261954 | 0.000343 |
| N-Acetylneuraminic acid | C19910 | HMDB0000230 | 7.2084 | 0.291482261 | 0.000347 |
| Hydroxyproline | C01157 | HMDB0000725 | 6.199178 | 0.272326611 | 0.000723 |
| Asparagine | C00152 | HMDB0000168 | 6.997922 | 0.364759624 | 0.000723 |
| Choline | C00114 | HMDB0000097 | 3.871205667 | 2.655437818 | 0.00077 |
| 5-Aminolevulinic acid | C00430 | HMDB0001149 | 6.855444333 | 11.59778499 | 0.000901 |
| Aminocaproic acid | C02378 | HMDB0001901 | 6.199178 | 0.439865583 | 0.000927 |
| Glycine | C00037 | HMDB0000123 | 6.642683333 | 0.455505103 | 0.001614 |
| S-Adenosylhomocysteine | C00021 | HMDB0000939 | 7.096539 | 0.464352044 | 0.001973 |
| L-Tyrosine methyl ester | C03404 | HMDB0029217 | 0.866922233 | 0.44310845 | 0.002417 |
| 2-Aminoisobutyric acid | C03665 | HMDB0001906 | 5.604677667 | 0.611239661 | 0.004062 |
| γ-Glutamylalanine | C03740 | HMDB0006248 | 8.088577667 | 2.024963844 | 0.004212 |
| Argininosuccinic acid | C03406 | HMDB0000052 | 8.947517 | 3.782950262 | 0.004521 |
| Homoserine | C00263 | HMDB0000719 | 6.388783333 | 0.479793435 | 0.005187 |
| Glutamine | C00064 | HMDB0000641 | 6.891066667 | 6.7312594 | 0.005612 |
| Threonine | C00188 | HMDB0000167 | 6.388783333 | 0.515767591 | 0.007238 |
| 3-Methylhistidine | C01152 | HMDB0000479 | 6.997922 | 0.393057165 | 0.008445 |
| N-Methyl-aspartic acid | C12269 | HMDB0002393 | 7.505700333 | 2.265931734 | 0.008458 |
| N6-Acetyllysine | C02727 | HMDB0000206 | 6.216411 | 0.397240997 | 0.011633 |
| 2,3-Diaminopropionic acid | C03401 | HMDB0002006 | 7.612067 | 1.550108643 | 0.013431 |
| Aspartic acid | C00049 | HMDB0000191 | 7.82295 | 1.47817582 | 0.020436 |
| N-Isovaleroylglycine | | HMDB0000678 | 2.266511333 | 2.984509387 | 0.025706 |
| Alanylglutamine |  | HMDB0028685 | 6.713816667 | 2.388659858 | 0.028316 |
| Ornithine | C00077 | HMDB0000214 | 8.820966333 | 2.37178792 | 0.028396 |
| Citrulline | C00327 | HMDB0000904 | 7.225194 | 2.477272516 | 0.028834 |
| β-Alanine | C00099 | HMDB0000056 | 6.216411 | 0.694669379 | 0.031252 |
| Cystathionine | C02291 | HMDB0000099 | 8.523706 | 1.087902432 | 0.031953 |
| Betaine | C00719 | HMDB0000043 | 4.052344333 | 1.449551586 | 0.032174 |
| Cysteine | C00083 | HMDB0000574 | 6.388783333 | 0.640212459 | 0.03497 |
| Lysine | C00047 | HMDB0000182 | 9.067205667 | 2.247726647 | 0.036696 |
| Alanine | C00041 | HMDB0000161 | 6.216411 | 0.726978973 | 0.038221 |
| 2-Phenylglycine |  | HMDB0002210 | 3.828905333 | 3.217274081 | 0.039554 |
| N-Acetylmethionine | C02712 | HMDB0011745 | 2.409211 | 1.523893079 | 0.041692 |
| Sarcosine | C00213 | HMDB0000271 | 6.216411 | 0.728781493 | 0.043775 |
| 4-Guanidinobutanoic acid | C01035 | HMDB0003464 | 6.855444333 | 1.352613698 | 0.047992 |
